# Genome-wide nucleosome mapping of *Plasmodium falciparum *reveals histone-rich coding and histone-poor intergenic regions and chromatin remodeling of core and subtelomeric genes

**DOI:** 10.1186/1471-2164-10-610

**Published:** 2009-12-16

**Authors:** Scott J Westenberger, Long Cui, Neekesh Dharia, Elizabeth Winzeler, Liwang Cui

**Affiliations:** 1 Department of Cell Biology ICND202, The Scripps Research Institute, 10550 North Torrey Pines Road, La Jolla, CA 92037, USA; 2 Department of Entomology, The Pennsylvania State University, 501 ASI Building, University Park, 16802 PA, USA

## Abstract

**Background:**

Epigenetic modifications of histones and regulation of chromatin structure have been implicated in regulation of virulence gene families in *P. falciparum*. To better understand chromatin-mediated gene regulation, we used a high-density oligonucleotide microarray to map the position and enrichment of nucleosomes across the entire genome of *P. falciparum *at three time points of the intra-erythrocytic developmental cycle (IDC) in vitro. We used an unmodified histone H4 antibody for chromatin immunoprecipitation of nucleosome-bound DNA.

**Results:**

We observed generally low nucleosomal occupancy of intergenic regions and higher occupancy of protein coding regions. In contract to the overall small fluctuation of nucleosomal occupancy in most coding regions throughout the IDC, subtelomeric genes encoding surface proteins such as *var *and *rif*, as well as some core chromosomal genes such as transcription factors, showed large changes in chromatin structure. Telomeres harbored a region with the highest nucleosomal occupancy of the genome and also exhibited large changes with higher nucleosomal occupancy at schizont stages. While many of these subtelomeric genes were previously shown to be modified by H3K9 trimethylation, we also identified some housekeeping genes in core chromosome regions that showed extensive changes in chromatin structure but do not contain this modification. tRNA and basal transcription factor genes showed low nucleosomal occupancy at all times, suggesting of an open chromatin structure that might be permissive for constitutively high levels of expression. Generally, nucleosomal occupancy was not correlated with the steady-state mRNA levels. Several *var *genes were exceptions: the *var *gene with the highest expression level showed the lowest nucleosomal occupancy, and selection of parasites for *var2CSA *expression resulted in lower nucleosomal occupancy at the *var2CSA *locus. We identified nucleosome-free regions in intergenic regions that may serve as transcription start sites or transcription factor binding sites. Using the nucleosomal occupancy data as the baseline, we further mapped the genome-wide enrichment of H3K9 acetylation and detected general enrichment of this mark in intergenic regions.

**Conclusions:**

These data on nucleosome enrichment changes add to our understanding of the influence of chromatin structure on the regulation of gene expression. Histones are generally enriched in coding regions, and relatively poor in intergenic regions. Histone enrichment patterns allow for identification of new putative gene-coding regions. Most genes do not show correlation between chromatin structure and steady-state mRNA levels, indicating the dominant roles of other regulatory mechanisms. We present a genome-wide nucleosomal occupancy map, which can be used as a reference for future experiments of histone modification mapping.

## Background

In eukaryotes, packaging of DNA into chromatin has profound effects on cellular processes that utilize DNA as the template, including transcription, replication, recombination and repair. The basic structural repeat unit of chromatin is the nucleosome, which contains ~150 bp of DNA wrapped in about 1.75 superhelical turns around a central histone octamer. The chromatin structure is complex and dynamic, changing through both covalent and non-covalent mechanisms [1]. Both histone tails and the globular domains are subject to a myriad of covalent modifications, including acetylation, methylation, phosphorylation, sumoylation, ubiquitylation, and ADP-ribosylation [2]. These modifications may directly affect the physical properties of chromatin, and also serve as a "histone code" that is read by other effector molecules [3]. Among non-covalent mechanisms, chromatin can be remodeled by ATP-dependent chromatin remodeling complexes and by incorporation of histone variants. The exchanges of histone variants such as H2A.Z and H3.3 with the canonical histones may influence the nucleosome stability and chromatin patterns [4,5]. Moreover, there also exists significant crosstalk among these chromatin-mediated epigenetic mechanisms. The advances in high throughput technologies such as ChIP-chip (chromatin immunoprecipitation - DNA microarrays) and "deep sequencing" (e.g., Illumina/Solexa technology) have enabled genome-wide profiling of histone modifications and variant histones. These studies have shown that specific histone variants and modifications are found to be associated with different regions of the genome to define active euchromatin and silent heterochromatin. Insights provided by these studies have significantly advanced our understanding of how chromatin organization regulates genome function.

As demonstrated in model eukaryotes, nucleosome positioning can be a major factor in regulating gene expression. Since nucleosomes generally impede transcription and high nucleosome occupancy is considered repressive, extensive remodeling and histone eviction occur during gene activation [6,7]. Consequently, nucleosome occupancy fluctuates during cell cycle or in response to environmental changes such as stress and activation [8,9]. Large-scale mapping of nucleosome occupancy has been performed in *Saccharomyces cerevisiae *[10-13], *Caenorhabditis elegans *[14,15], *Drosophila melanogaster *[16], and human [9]. These studies have revealed that promoters are often depleted of nucleosomes, and gene expression is correlated inversely with nucleosome occupancy at the promoter. Further, functional transcription factor binding sequences are also nucleosome-free regions [17]. Especially, nucleosome positioning around the active transcription start sites has a similar organization patterns in different eukaryotes [9,13,16]. These high-resolution nucleosome occupancy data also allowed the validation of computational models to predict nucleosome organization, which suggest the presence of intrinsic signals for nucleosome occupancy encoded across the genome [18,19].

The malaria parasite *Plasmodium falciparum *causes over one million deaths each year. Over the last two decades, our knowledge of the malaria parasites at the molecular level has expanded substantially, particularly with the completion of the genome-sequencing project [20]. Microarray analysis has revealed patterns of changes in mRNA levels throughout the intra-erythrocytic developmental cycle (IDC) of this parasite [21,22]. While regulation of transcription in the malaria parasite is poorly understood, the conservation of many chromatin-modification factors in the genome underlines the significance of epigenetic mechanisms in this parasite [23]. The malaria parasite genome encodes four canonical and four variant histones and nuclear DNA is organized in typical eukaryotic nucleosomes [24,25]. Whereas *Plasmodium *chromosomes are not highly condensed during its cell cycle, indirect evidence suggests the existence of heterochromatin [26]. A number of modifications such as acetylation and methylation have been found on the histones [25,27] and their roles in transcription regulation appear to be evolutionarily conserved. For example, the H3K9 acetylation, conferred by the histone acetyltransferase (HAT) GCN5, is associated with active genes, whereas the heterochromatin marker H3K9 trimethylation (H3K9me3) is associated with silent genes [28-30]. Another prominent example of epigenetic regulation in *Plasmodium *is the regulation of the mutually exclusive expression of the ~60 *var *genes, which mediate antigenic switching [26]. Silencing of the telomeric clusters of *var *genes requires the Sir2 histone deacetylase [31,32]. While research on epigenetics in the malaria parasite is still in its infancy, there is a clear indication that chromatin remodeling represents an important mechanism of gene regulation and high throughput technologies such as ChIP-chip are also feasible for the extremely AT-rich genome.

Expanding on our previous success of mapping parasite histone marks using ChIP and low-density microarrays, we report here a high-density atlas of the nucleosome occupancy through the IDC of *P. falciparum *using a custom whole-genome tiling microarray.

## Results

### Genome-wide nucleosomal occupancy in P. falciparum

We analyzed the genome-wide nucleosomal occupancy in synchronized in vitro cultures of *P. falciparum *for three time points from ring, trophozoite and schizont stages covering the IDC. Parasites were treated with formaldehyde to cross-link DNA-binding proteins to the genomic DNA and nucleosomal DNA was then immunoprecipitated using an antibody to histone H4. The purified DNA was hybridized to a custom whole-genome tiling array and compared with 3D7 genomic DNA hybridization data to determine relative enrichment of histones genome-wide.

**Figure 1:**
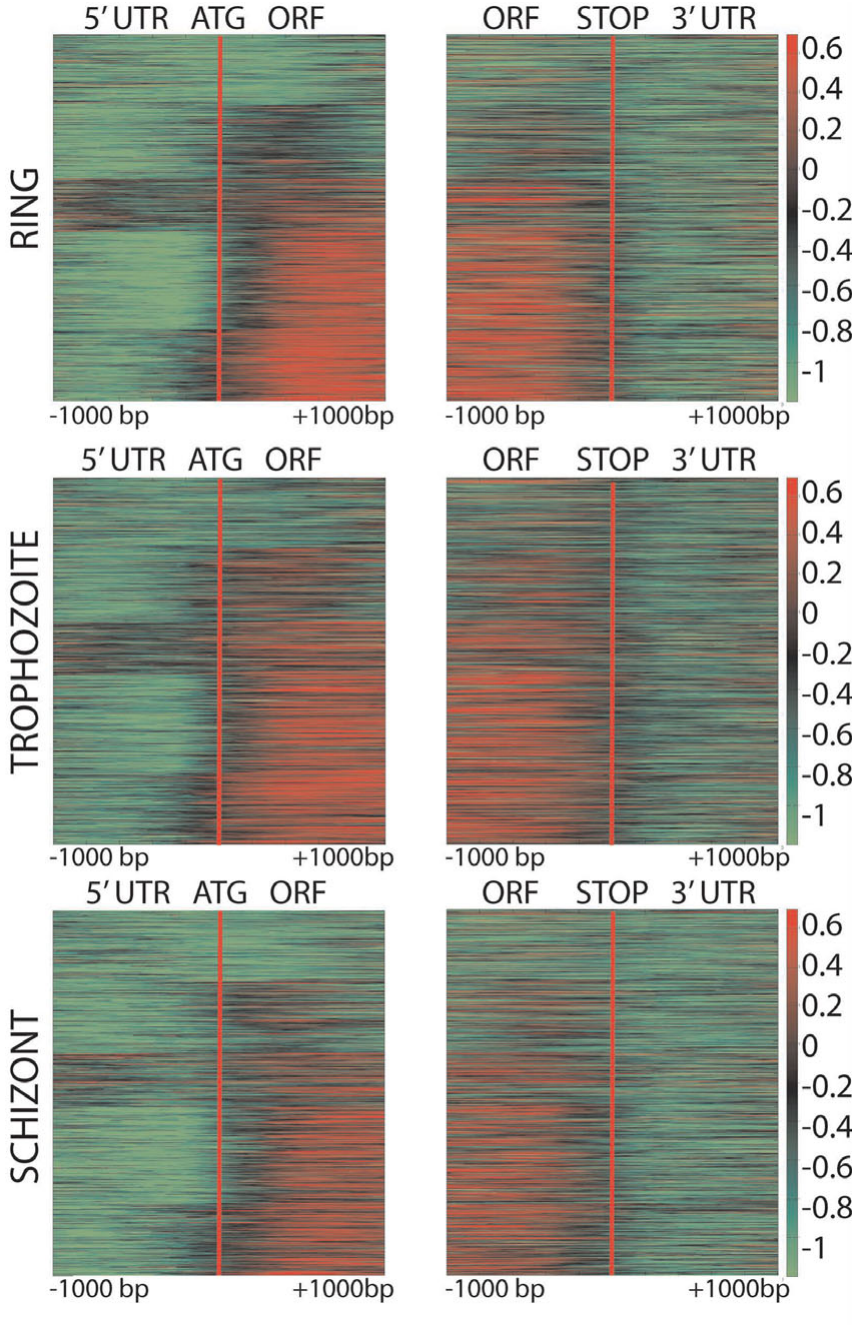
**Nucleosome enrichment correlates with protein coding region for most genes**. The patterns of log_2 _ratio of H4 ChIP for the 1 kb region upstream and downstream and inside of the open reading frame (ORF) for all genes at ring (top row), trophozoite (middle row), and schizont (bottom row) stages were grouped into five sets using K-means clustering. The red line indicates the start and end of the ORF.

Significant differences in nucleosomal enrichment were found throughout the genome. For the purposes of our analysis, we divided the parasite chromosomes into different domains. We defined telomeric regions as those regions from the end of chromosome telomeric repeats to the beginning of the coding region of the first gene, and subtelomeric regions as those from the start of the first gene to the first housekeeping genes with syntenic orthologs in *P. vivax *or *P. yoelii*. The rest of the regions were defined as core chromosome regions. Therefore, subtelomeric genes include members of multigene families involved in antigenic variation such as *var, rifin, stevor*, two transmembrane (2-TM), erythrocyte binding antigens (EBA), merozoite surface proteins (MSP), PHIST, and other species-specific genes. To determine whether nucleosomal occupancy is different between coding and intergenic regions, we calculated the average log_2 _ratio of nucleosomal enrichment for all genes and exons in the genome (Additional file 1). The average nucleosome enrichment of all coding regions was positive, with some fluctuations throughout the IDC ranging from 0.1 to 0.24 log_2 _ratio (Table 1), indicating that coding regions were generally enriched with nucleosomes. In contrast, non-coding core chromosomal regions had an average log_2 _ratio of -1, indicating low nucleosomal occupancy, and were less variable than core coding regions. The exception was the non-coding subtelomeric regions, which had positive log_2 _ratios, indicating nucleosome enrichment and tight packaging of telomere associated repeat elements (TAREs). To visualize genome-wide correlations of coding regions and nucleosome enrichment boundaries, we examined the regions 1 kb upstream and downstream of the putative ATG translation start codon and 1 kb upstream and downstream of the stop codon for all genes. We clustered the nucleosome enrichment data for these regions into five K-means clusters to identify different patterns. For most genes, the nucleosome enrichment began within 200 bp of the putative coding region (Figure 1).

**Table 1:** Mean nucleosome enrichment values and Pearson correlation of nucleosome patterns between different stages.

		Mean Log_2 _ratio of probes ≤10				Pearson Correlation Coefficient		
		
		Change	Ring	Trophozoite	Schizont	Ring vs Trophozoite	Ring vs Schizont	Trophozoite vs Schizont
Whole Genome	All Probes	0.14	-0.14	0.00	-0.12	0.70	0.89	0.57
	Coding	0.21	0.10	0.24	0.04	0.70	0.85	0.56
	Non-coding	0.20	-0.62	-0.50	-0.42	0.70	0.93	0.59

Core Regions	All Probes	0.19	-0.11	-0.01	-0.20	0.71	0.89	0.55
	Coding	0.22	0.13	0.22	0.00	0.72	0.85	0.55
	Non-coding	0.13	-0.76	-0.62	-0.73	0.69	0.93	0.55

Sub-telomere	All Probes	0.46	-0.40	0.01	0.06	0.69	0.93	0.60
	Coding	0.65	-0.19	0.46	0.34	0.64	0.89	0.54
	Non-coding	0.40	-0.56	-0.34	-0.17	0.71	0.94	0.63

Internal *var *clusters	All Probes	0.77	-0.50	0.27	0.23	0.67	0.93	0.60
	Coding	1.03	-0.37	0.66	0.51	0.59	0.93	0.49
	Non-coding	0.61	-0.62	-0.08	-0.01	0.72	0.94	0.67

Telomere	All Probes	1.50	0.43	-0.05	1.45	0.63	0.93	0.52

While most genes demonstrated nucleosome enrichment in the coding regions, some genes showed a divergence from this pattern. The first set of 1075 genes showed low nucleosomal occupancy throughout the coding regions, which includes most tRNAs, many (26 of 44) 40S and 60S ribosomal protein subunits, RNA polymerase II subunits and associated basal transcription factors such as TFIIS, as well as U6 snRNA and most LSM-domain containing small nuclear ribonucleoproteins (snRNPs) involved in ribosomal RNA maturation and mRNA splicing. The second set of 1104 genes appeared to cluster those genes with small 5' exons, visualized by the small red peak following the start of the gene. This set includes most *var, rifin *and *stevor *and some other subtelomeric variant surface antigens (VSAs), which have small 5' exons with secretory signals and generally show lower nucleosome enrichment in their coding regions than core chromosomal genes (Table 1). The third set of 918 genes had peak nucleosome enrichment upstream of the putative ATG, which includes some genes that are close to another upstream coding region and genes for which there are no probes in the upstream coding regions, resulting in log_2 _ratios of zero for missing data. The remaining sets four and five with 2559 genes include the vast majority of core chromosomal protein-coding genes.

**Figure 2:**
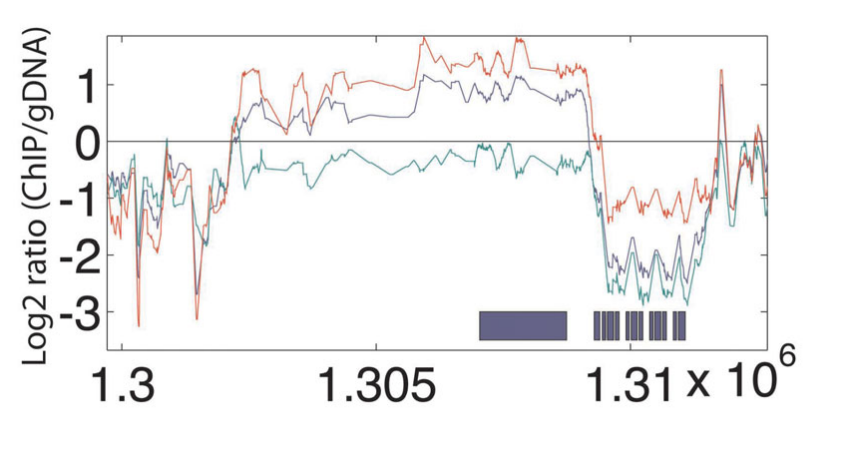
**New gene predicted by nucleosome enrichment pattern**. The nucleosome enrichment pattern between genes *PFF1510w *and *PFF1515c *on chromosome 6 suggests the presence of a non-annotated gene. The blue boxes represent an Evigan gene model predicted by PlasmoDB. The histone pattern suggests a much larger first exon, or additional large exons upstream of the gene model with a putative nucleosome-free TSS around 13104500 bp. The x-axis indicates the base pair position on the chromosome.

Since nucleosome enrichment appeared to correlate with the coding regions, we searched for regions of high nucleosome enrichment that did not overlap annotated genes. Using this method we identified some additional non-annotated exons and a few genes that have recently been annotated following the *P. falciparum *genome re-annotation, adding more evidence to the existence of these protein-coding genes. A new putative gene-coding region on chromosome 6 was identified based on the pattern of nucleosome enrichment (Figure 2). Gene prediction algorithms used by PlasmoDB support this new gene model.

### Nucleosomal positioning

Individual nucleosome position appeared to be fixed for most coding regions, since the nucleosome mapping pattern did not change for most genes throughout the IDC, showing that coding regions are tightly bound by closely spaced nucleosomes. In some coding regions, less distinct nucleosome peaks implied a delocalized nucleosome arrangement, but this pattern was similar throughout the IDC. Intergenic regions had some peaks of fixed nucleosome positions that also maintained their position specificity throughout the IDC, while overall nucleosome enrichment remained low.

To examine the hypothesis that nucleosome enrichment is determined by DNA sequence, we examined the distribution of log_2 _ratios for 25mer probes with different numbers of G or C nucleotides (GC bins 1-25) in various regions of the genome (Additional file 2). The average GC content of probes in various regions ranged from 5 to 7 GCs per 25mer oligonucleotide (Additional file 3). The highly AT-rich genome of *P. falciparum*, with higher AT content in the intergenic regions than coding regions, results in higher average probe GC content in coding than non-coding regions. There was a higher average log_2 _ratio of nucleosome enrichment in regions with higher GC content. This effect was more pronounced in intergenic regions, while coding regions showed a more equal log2 ratio distribution across GC bins. However, even GC-rich non-coding regions showed lower log2 ratios of nucleosome enrichment than highly AT-rich coding regions. Therefore, while the highly AT-rich intergenic regions may be predisposed to lower nucleosomal occupancy, DNA sequence does not appear to be the primary determinant of chromatin nucleosome enrichment, which may be specifically coordinated with the delineation of coding versus non-coding regions.

### Telomeric changes in chromatin structure

**Figure 3:**
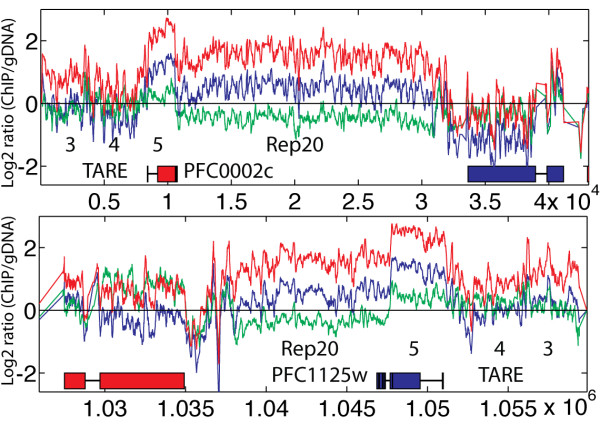
**Extensive changes of nucleosome occupancy at the telomeres**. Nucleosomal enrichment changes represented by chromosome 3 left and right telomeres and boundary element regions. Lines indicate average log_2 _ratio of H4 ChIP divided by genomic DNA hybridization intensity over a window of 150 bp: blue = ring, green = trophozoite, red = schizont. Blue (Red) genes are encoded on the top (bottom) strand. The x-axis indicates the base pair position on the chromosome.

The most striking changes in nucleosome enrichment took place in telomeric and subtelomeric regions, indicating large-scale chromatin remodeling throughout the IDC. The telomeric regions closest to the chromosome ends displayed moderate nucleosome enrichment in rings, lowest in trophozoites, and highest in schizonts (Figure 3). Telomeres are comprised of the telomere repeat itself, followed by a series of TAREs. Internal to the TAREs is the repetitive region known as Rep20 of 5-28 kb (mean = 12 kb) stretching from the last *var *gene to the TARE5 [33]. We identified a distinct peak of much higher nucleosome enrichment within the telomeric region of 1500 bp corresponding to the TARE 5 [34], previously described as 1.4-2 kb of the 12-bp repeated sequence ACTAACA(T/A)(C/G)A(T/C)(T/C) [35-37]. Some paralogous *P. falciparum*-specific genes are annotated in these regions (*PFC1125w, PFC0002c, MAL8P1.335, PFD1250w*). They are the most telomere-proximal open reading frames annotated on the chromosomes and are likely not true protein-coding genes. The TARE 5 regions had the highest nucleosomal enrichment of any region in the genome. Nucleosome enrichment decreased but retained the same pattern in the more telomere-proximal TAREs. This observation suggests that TARE 5 may function as a sequence boundary element separating telomeres from coding regions. The Rep20 element has been shown to confer improved association and segregation with nuclear chromosomes to transfected plasmids [35], indicating that this region associates with proteins responsible for chromosome segregation. The high nucleosome enrichment in schizonts may represent condensation of telomeric sequences in late schizont stage when DNA is being replicated and packaged into daughter cells.

### Different patterns of nucleosomal occupancy dynamics

**Figure 4:**
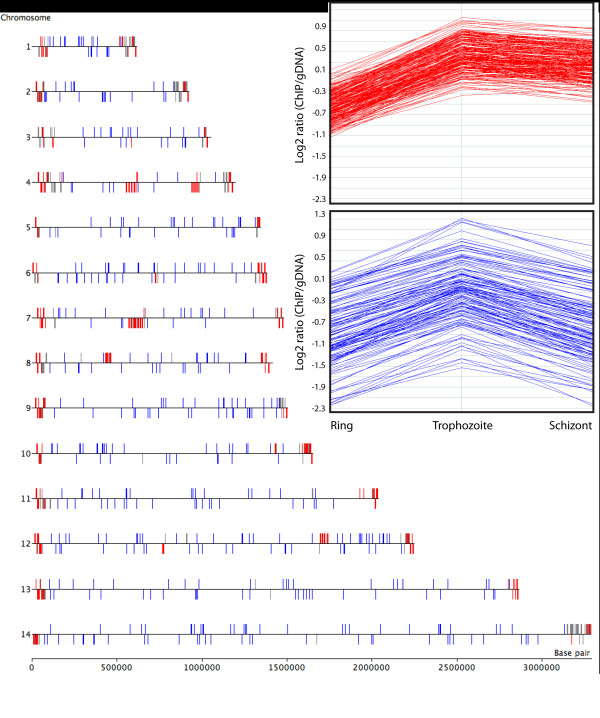
**Genome-wide nucleosome occupancy analysis shows extensive changes for subtelomeric and core chromosomal genes**. Subtelomeric genes with >1.5-fold changes (Δlog_2 _ratio>0.6) in nucleosome occupancy during IDC are shown in red. Grey genes indicate those enriched with H3K9m3 modification [38] but do not show large changes in nucleosome enrichment. Core chromosomal genes with large changes in nucleosome enrichment during ring and trophozoite stages but with greater decrease in schizont stage are shown in blue. Inset boxes show the pattern of change in nucleosome enrichment for the selected genes throughout the IDC.

**Figure 5:**
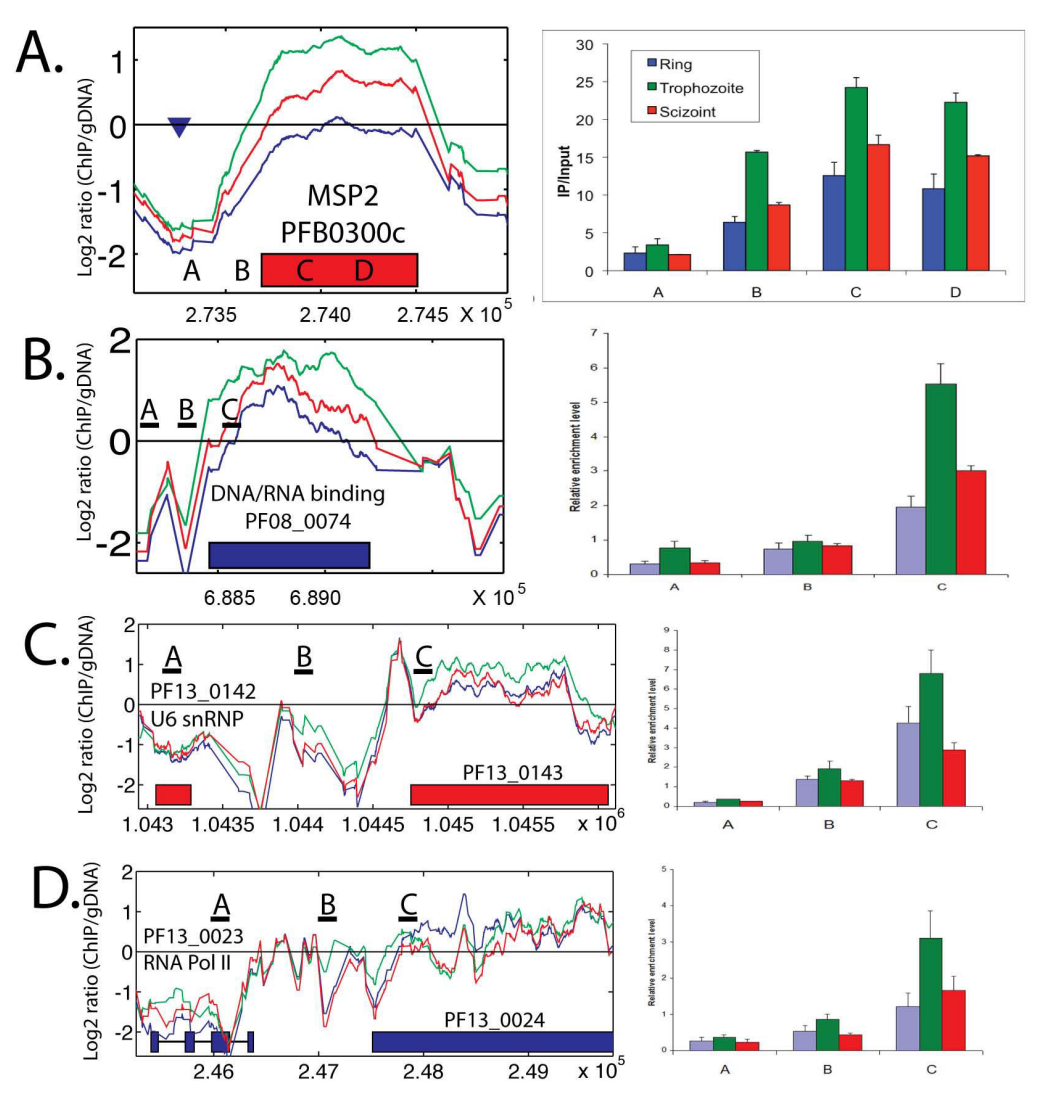
**Different types of nucleosome occupancy change in the *Plasmodium *genome**. Shown here are representatives of each type. The left panel was generated from the microarray data. Lines indicate average log_2 _ratio of H4 ChIP divided by genomic DNA hybridization intensity over 150 bp windows: blue = ring, green = trophozoite, red = schizont. Blue (Red) genes are encoded on the top (bottom) strand. The x-axis indicates the base pair position on the chromosome. The right panel shows Q-PCR results to validate the microarray results. The relative enrichment level represents the 2^-ΔΔCt ^value obtained by comparing anti-H4 ChIP DNA with input DNA and normalizing the data with the control gene *MAL8P1.36 *which had relative stable nucleosome occupancy through IDC.

Comparison of nucleosomal occupancy at three time points of the IDC showed great temporal changes for many genes and chromosomal regions (Figure 4). Whereas the overall fluctuation of the average nucleosome enrichment of all coding regions was small (Table 1), subtelomeric coding regions were highly variable. While most of the genes (440) with >1.5 fold changes in nucleosome enrichment throughout IDC were distributed in the subtelomeric regions, there were also many in the core chromosomal regions (Figure 4). In contrast to the telomere regions, subtelomeric genes showed a distinctly different pattern with low nucleosomal occupancy in ring stage, and higher enrichment in trophozoite and schizont stages (Figure 4, top inset). We identified another pattern of nucleosome enrichment changes in core chromosome genes similar to that of the subtelomeric VSAs with low nucleosome enrichment in rings and highest in trophozoites, but these genes showed a greater reduction in nucleosome enrichment in schizonts, whereas the VSAs maintained a higher nucleosome enrichment in schizonts. Genes with this pattern (294 genes) in core chromosomal regions (Figure 4, bottom inset) include a large number of genes involved in transcription. Their nucleosomal enrichment ranged from very low to high, but they all shared the pattern of highest enrichment in trophozoites. Only a few core chromosomal genes with this pattern were also enriched in H3K9me3 (Additional file 4, Table 2). It is interesting to note that many of these genes that are subject to chromatin remodeling encode proteins that carry out histone modifications, remodel chromatin, or bind DNA to regulate transcription (Additional file 5, Table 3). The nucleosomal occupancy patterns were confirmed for *MSP2 *(*PFB0300c*) and a DNA/RNA binding protein (*PF08_0074*) using quantitative PCR (Q-PCR) (Figure 5A and 5B).

**Table 2:** Core chromosome genes with H3K9me3 modification. H4 change is the Δlog2 ratio (H4 ChIP/gDNA) over three time points.

Gene	Description	H4 Change
PFA0420w	Conserved *Plasmodium *protein, unknown function	1.42
PFL0145c	high mobility group protein	1.34
PF10_0355	Erythrocyte membrane protein, putative	0.94
MAL8P1.71	RNA binding protein, putative	0.75
PFD0100c	Surface-associated Interspersed gene SURFIN 4.1	0.71
MAL8P1.137	conserved *Plasmodium *protein, unknown function	0.54
PF14_0390	conserved *Plasmodium *protein, unknown function	0.47
PF13_0168	CPW-WPC family protein	0.46
PFE0160c	Ser/Arg-rich splicing factor, putative	0.46
PFL1085w	transcription factor with AP2 domain(s)	0.46
PF10_0159	glycophorin-binding protein 130 precursor	0.43
PFC0110w	Cytoadherence linked asexual protein 3.1	0.41
PFC0105w	serine/threonine protein kinase, putative	0.41
PFC0120w	Cytoadherence linked asexual protein, 3.2	0.38
PFL2075c	conserved *Plasmodium *protein, unknown function	0.37
PF10_0356	liver stage antigen-1	0.34
PF13_0191	conserved *Plasmodium *protein, unknown function	0.30
PF11_0422	conserved *Plasmodium *protein, unknown function	0.25

H3K9m3 modification status was determined by Salcedo-Amaya et al. [38]

**Table 3:** Transcription and chromatin-related genes with extreme changes in nucleosome enrichment, highest in trophozoites.

Gene	Description	H4 Change
PFL0145c	high mobility group protein	1.34
PF10_0217	pre-mRNA splicing factor, putative	1.22
MAL7P1.78	Transcription factor IIA, alpha/beta subunit	1.03
PF11_0266	small nuclear ribonucleoprotein D1, putative	1.00
PF08_0074	DNA/RNA-binding protein Alba, putative	0.99
PFI1835c	Nifu-like protein, putative	0.98
PF14_0411	small nuclear ribonucleoprotein, putative	0.95
PF14_0242	arginine n-methyltransferase, putative	0.94
PF10_0227	HORMA domain protein, putative	0.93
PFF0565c	EF-hand domain hypothetical protein, conserved	0.87
PFF0760w	RNA and export factor binding protein, putative	0.87
PFC1050w	tudor domain conserved *Plasmodium *protein	0.86
PF07_0057	transcription elongation factor s-ii, putative	0.83
MAL8P1.72	high mobility group protein	0.82
PFD0685c	chromosome associated protein, putative	0.78
PF14_0533	transcription factor with AP2 domain(s)	0.75
PFL1705w	RNA binding protein, putative	0.74
PF10_0232	Chromodomain-helicase-DNA-binding protein 1	0.72
PFD0795w	Histone acetyltransferase, putative	0.69
PF13_0318	RNA-binding protein, putative	0.69
PFC0610c	zinc finger protein, putative	0.68
PFD0985w	transcription factor with AP2 domain(s)	0.66
MAL7P1.157a	RNA binding protein, putative	0.63
PFE0520c	topoisomerase I	0.63
PFC0130c	RNA binding protein, conserved	0.62
PF10_0214	RNA binding protein, putative	0.62
PFL1085w	transcription factor with AP2 domain(s)	0.46

H4 change is the Δlog2 ratio (H4 ChIP/gDNA) over three time points.

Depending on the dynamics of nucleosome enrichment in different developmental stages, genes can be organized into distinct categories. Some genes involved in basal transcription machinery, ribosomal RNA maturation and mRNA splicing were always nucleosome-free (Additional file 6). Genes for U6 snRNA LSM protein (PF13_0142) and RNA polymerase II (PF13_0023) were confirmed to have low nucleosomal occupancy less than or similar to intergenic regions, and much lower than adjacent genes (Figure 5C and 5D). The maintenance of an open chromatin conformation may allow for high levels of constitutive expression of these genes. Numerous hypothetical genes with similarly low nucleosome enrichment may also play a role in these processes. In addition to the genes found in TARE 5 telomeric regions described above, we found several genes that displayed nucleosomal occupancy changes similar to those seen for telomeres. These genes encode the Pf11-1 protein PF10_0374, S-antigen, Liver Stage Antigen 1, interspersed repeat antigen, Maurer's cleft two transmembrane protein 1.1, *Plasmodium *MYXSPDY repeat protein, two glycophorin binding protein homologous proteins, a highly variable hypothetical protein PF10_0351, and several conserved hypothetical proteins (Additional file 7). All these genes displayed a very large increase in nucleosome enrichment (> 0.7 log2 ratio) from trophozoite to schizont stages and intermediate levels in rings, placing them in the top 20% of all genes ranked by nucleosomal occupancy change throughout the IDC. These genes contain highly repetitive regions, and may be regulated by the same chromatin remodeling machinery that is targeted to the highly repetitive telomere sequences. It is interesting to note that many of these genes encode highly variable surface proteins, and five of them are found in close proximity on chromosome 10. Another small group of genes including an amino transferase, leucine-rich repeat protein 2 and a PPPDE peptidase showed yet a different pattern with highest nucleosome enrichment in rings, lowest in trophozoites, and a slight increase in schizonts (Additional file 8).

Interestingly, the non-coding RNA types showed different patterns of nucleosome enrichment. tRNA genes have the lowest nucleosomal occupancy of any set of genes, lower than the average for intergenic regions, indicating that their chromatin structure is different from those of protein-coding RNAs. This nucleosome-free status of tRNAs may be due to their essential role in translation and high abundance in the cell, or may be a specific feature of RNA polymerase III (Pol III) transcribed regions. Another Pol III transcript, 5S rRNAs, had much lower nucleosome enrichment than other rRNAs throughout the IDC and showed a different pattern of enrichment changes. 5.8S rRNAs showed higher nucleosome enrichment in rings relative to 18S and 28S rRNAs, with little change in trophozoites and a large decrease in schizonts. The 18S and 28S rRNA loci showed a large increase from rings to trophozoites and decrease again in rings. This 18S and 28S rRNA pattern is similar to many subtelomeric genes. While the loci on chromosomes 1 and 5 are subtelomeric and thus might be expected to behave similarly to other subtelomeric genes, the chromosome 7 locus is not subtelomeric, and therefore this pattern may be specific to rRNA loci and not a byproduct of their subtelomeric localization.

### Comparison of cDNA start sites and nucleosome free-regions

To test the hypothesis that nucleosome-free regions may exist at transcription start sites (TSS) as has been shown in other organisms, we examined the nucleosome enrichment at over 2600 putative transcription start sites annotated by the Malaria Full-Length cDNA database http://fullmal.hgc.jp/index_ajax.html[36]. We found a large distribution of nucleosome enrichment values at these sites (Additional file 1). The median value for these regions was between -0.5 and -0.7 for the various stages (Additional file 1), which was higher than the mean for core chromosomal non-coding regions (Table 1). However, some of these TSS are predicted inside coding regions, which had median log2 ratios of around 0.2. We also predicted absolute minima of a 150 bp window running mean of the log2 ratio of nucleosome occupancy in the upstream intergenic regions of all genes (Additional file 1). Multiple, additional local minima - nucleosome-free regions were also observed in many intergenic regions. While some cDNA sequences did appear to begin at intergenic local minima of nucleosome enrichment (Additional file 1), consistent with the hypothesis, many others were found at intergenic local peaks of nucleosome enrichment. Therefore, our dataset is not completely consistent with the hypothesis that TSSs correlate with nucleosome-free intergenic regions for all genes. The caveat is that given low probe coverage for intergenic regions, their chromatin structure may not be accurately represented in our data. Besides, the cDNA sequence dataset is also incomplete, providing TSS predictions for about half of the genes and further cDNA sequencing may show better correspondence with our nucleosomal occupancy data. Furthermore, many *P. falciparum *genes have been shown to use multiple TSSs, which may obscure the minima of nucleosomal occupancy within the intergenic regions. Additionally, nucleosomal occupancy may differ at other points of the life cycle not represented in our dataset, during which some genes are most highly expressed. Taken together, transcription of some genes may be initiated at these nucleosome-free regions (NFRs) (Additional file 1), while others may have alternative requirements for chromatin structure at the TSS.

### Correlation of nucleosome enrichment with gene expression

Overall, changes in nucleosome enrichment were not correlated with gene expression for the majority of genes in the genome. We calculated the Pearson correlation coefficients between the log2 ratios of nucleosome enrichment and gene expression values from sorbitol-synchronized *in vitro P. falciparum *culture analyzed previously [22]. There was no genome-wide pattern of correlation or anti-correlation with expression throughout the IDC. This implies that for most genes, their expression is not regulated by, nor does it result in changes in the nucleosome enrichment of the gene coding regions. However, particular subsets of genes did show interesting patterns and correlations between expression and nucleosome enrichment.

We identified 851 genes with high expression (>500 units) [37] at some time point of the IDC, and showed minimal changes in nucleosomal occupancy (Δlog_2 _ratio < 0.5). The nucleosomal occupancy of these genes ranged from nucleosome-free (log_2 _ratio < -1.5) to highly occupied (log_2 _ratio >0.7). This group includes genes upregulated in merozoite and early ring stages such as merozoite-associated tryptophan-rich antigen, PTRAMP, RESA, PHISTs, and other genes involved in host cell remodeling. It also includes genes upregulated in late schizont stage involved in gliding motility and invasion of red blood cells such as RAP1, RAP3, RBP2a, RBP2b, RBP3, apical sushi protein (ASP), MSP3, MSP9, EBA-140, CLAG3.2, AMA1, and SPATR. This indicates that these genes involved in merozoite development and invasion are not regulated at the level of nucleosomal occupancy, and their high expression is not associated with large changes in nucleosomal occupancy.

In comparison, 315 genes showed large changes in nucleosomal occupancy (Δlog_2 _ratio >0.7, 2-fold), but are not highly expressed (<200 units) at any time point in the IDC [37]. These include numerous subtelomeric members of multigene families of antigenic surface protein genes, such as *pfemp1, surfins, rifins *and *stevors*, of which some members are highly expressed and subject to the general nucleosomal occupancy changes regulating all subtelomeric regions. These genes showed low nucleosome enrichment in ring stages when expression of these genes is highest, and were nucleosome-enriched in trophozoites and schizonts. In contrast, most low-expressing genes in core chromosome regions (76 of 128) with large changes in nucleosomal occupancy showed the opposite pattern of high nucleosomal occupancy in rings and low in trophozoites and schizonts (Additional file 8). This set of 120 genes also includes cysteine repeat modular protein 2, transcription elongation factor S-II, leucine-rich repeat proteins LRR2 and LRR10, and UbiE-like methyltransferase. These patterns suggest the possible existence of two kinds of chromatin remodeling machinery acting with opposing results targeted to different chromosomal regions. However, chromatin remodeling at these genes did not appear to affect their mRNA levels during the IDC.

### Nucleosome occupancy changes of the var genes

**Figure 6:**
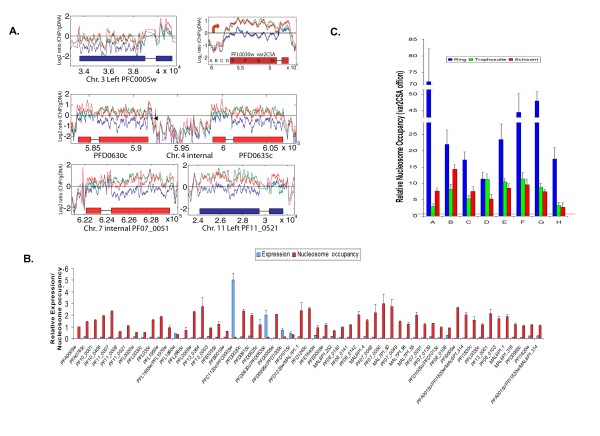
**Correlation of gene expression with the levels of nucleosomal occupancy among *var *genes**. A. Microarray results of nucleosome occupancy in representative *var *genes. The *var *gene *PFC0005w *displayed the lowest nucleosomal occupancy of all *var *gene, whereas *PFD0630c *and *PFD0635c *had the next lowest nucleosomal occupancy. *PF07_0051 *(internal) and *PF11_0521 *(subtelomeric) represent *var *genes with higher nucleosome occupancy. The x-axis indicates the base pair position on the chromosome. **B. The relative expression level (red) and nucleosomal occupancy level (blue) of individual *var *genes in an unselected parasite population**. The var genes' expression levels had a inverse correlation with the levels of nucleosomal enrichment (Pearson correlation *R*^2 ^> 0.90). **C. Relative nucleosomal occupancy of *var2CSA *gene in CSA-unselected (silent) versus CSA-selected (active) parasites**. Relative nucleosome enrichment levels for *var2CSA*-off versus *var2CSA*-on parasites were determined during three developmental stages. The locations of the primers to map these genes are marked with A to H in Figure 6A.

Most genes contained in the subtelomeric regions are members of multigene families of VSAs such as *vars *and *rifins*. The nucleosome enrichment pattern was the same for most *var *and *rifin *pseudogenes and numerous other subtelomeric genes of unknown functions. We found a few exceptions that despite their subtelomeric locations showed minimal change in chromatin structure. For example, only 10 of 57 *var *genes, all pseudogenes, and two of 40 *stevor *genes, and two of 176 *rifins *showed little change in nucleosome enrichment (Δlog_2 _ratio < 0.5). The *var *gene with the lowest nucleosomal occupancy is PFC0005w (Figure 6A). The *var *genes with the second and third lowest nucleosomal occupancy are *PFD0630c *and *PFD0635c*, which had much higher nucleosome enrichment than *PFC0005w *but lower than all others. The low nucleosomal occupancy of these subtelomeric genes in ring stages revealed a chromatin structure that might be highly permissive for transcription and correlated with their highest expression. Real-time RT-PCR analysis of RNA extracted from the same sample confirmed that *PFC0005 *was the dominant *var *gene expressed in this parasite population (Figure 6B), and Q-PCR analysis of immunoprecipitated DNA also confirmed that this gene had the lowest nucleosomal occupancy of all *var *genes (Additional file 1). Similarly, *PFD0630c *and *PFD0635c *were shown to have the second highest levels of RNA expression by real-time RT-PCR, about half that of *PFC0005w *(Figure 6B). In the unselected parasite population, *var2CSA *was generally silent (Figure 6A). To further confirm that nucleosome occupancy is inversely correlated with *var *gene expression, we used chondroitin sulfate A (CSA) panning to select parasites with *var2CSA *expression. When *var2CSA *was active, we found a 10-70-fold decrease in nucleosome occupancy at the *var2CSA *locus (Figure 6C). Interestingly, the small, conserved exon 2 of *var *genes shows a different nucleosome enrichment pattern with the highest enrichment in schizonts, medium in rings and lowest in trophozoites. Exon 2 had higher log_2 _ratio (average = 0.55) of nucleosome enrichment than exon 1 in ring stages, but this difference was reversed in trophozoite stages where exon 1 was higher than exon 2 (average difference = 0.5) (Additional file 1). We found that a subtelomeric *var *gene (*PF11_0521*) and an internal *var *gene (*PF07_0051*) showed more equal nucleosomal enrichment between the two exons (Figure 6). This difference may be linked to the function of intrinsic *var *gene intron promoter activity. Other genes for surface proteins, such as the related *PfEMP3 *gene, showed a completely different chromatin structure throughout the IDC.

This pattern of nucleosome enrichment changes at *var *genes was also found among other VSA genes. For the 129 *rifin *genes, they were histone-poor in ring stages (log_2 _ratio = -0.64) but more histone-rich in trophozoites and schizonts (log_2 _ratio = 0.30 and 0.14). The nucleosome enrichment pattern for *var *genes was slightly different from that of *rifins *with average nucleosomal occupancy in rings (log_2 _ratio = -0.22) and much higher than average nucleosomal occupancy in trophozoites and schizonts (log_2 _ratio = 0.8 and 0.72). To identify genes with similar chromatin remodeling patterns, we used *var *and rifin genes as seeds to search for genes with highly correlated changes (>0.95 Pearson coefficient) in nucleosomal enrichment throughout the IDC. This identified a set of 226 genes with large changes in nucleosome enrichment (Δlog_2 _ratio > 0.6). These genes are found exclusively in subtelomeric regions or internal *var *gene clusters (Figure 4). Most of these genes were also previously described as enriched in the histone H3K9me3 modification, a marker of heterochromatin [38].

### H3K9 acetylation modification pattern

The variation in nucleosomal occupancy observed using the unmodified histone H4 antibodies was also visible using histone H3K9 acetylation (H3K9ac) antibodies. Therefore, to properly determine regions enriched for H3K9 acetylation, it is necessary to normalize the data relative to the unmodified histone H4 hybridization pattern, rather than to genomic DNA. When histone H3K9ac ChIP DNA was normalized relative to genomic DNA, the pattern followed the H4 pattern of nucleosomal occupancy with slightly higher peaks (data not shown). Therefore, the distribution of H3K9ac generally followed the background distribution of nucleosomes. Normalization to the H4 ChIP pattern resulted in higher signals in the intergenic regions, perhaps due to lower, local nucleosomal occupancy. Very distinct spikes were observed in regions of the highest nucleosomal occupancy. This indicates that while the nucleosomal occupancy was generally lower in intergenic regions, the majority of the histones in the intergenic regions contained the H3K9ac modification. The H3K9ac enrichment appeared in all intergenic regions and did not appear to be correlated with changes in mRNA levels. These data confirmed the findings of Salcedo-Amaya et al. who also found that H3K9ac was enriched in all intergenic regions, and only appeared higher for very highly expressed genes in schizont stages [38].

## Discussion

The association of nucleosomes with coding regions and their relative absence from intergenic regions is distinctly different from the findings in model eukaryotes, where nucleosomes are usually absent only at promoters and intergenic regions are condensed. In *P. falciparum *genome, this may be the result of the extreme AT-richness in the intergenic regions. Since poly-A tracts induce bending of the DNA helix [39] and this bending tends to exclude nucleosomes from these regions [40], the high AT content (95%) of intergenic regions of *P. falciparum *may selectively exclude histones from these regions. While model organisms generally have small nucleosome-free regions observed at TSSs [11,41], our analysis using the *P. falciparum *full-length cDNA data was inconclusive. Instead, a more extensively nucleosome-free region may be present in the intergenic regions of the parasite genome allowing easy access to RNA polymerase machinery and transcription factors. This may be partially related to the diverse transcription initiation sites found in many *P. falciparum *genes [42]. Yet, in spite of the lower nucleosomal occupancy at the intergenic regions, there were spikes of modified histones in the intergenic regions, which may play additional roles in gene expression regulation. The higher nucleosomal occupancy in coding regions of most genes may provide a means to safeguard against spurious transcription initiation in the coding region of the gene.

In model eukaryotes, gene expression is positively correlated with the nucleosome occupancy at the promoter regions. In contrast, the intergenic putative promoter regions of *P. falciparum *showed little or no change in nucleosomal occupancy, and generally remained nucleosome-free, compared to the large changes observed in coding regions. Comparing the nucleosomal occupancy of the coding regions, we found no general correlation with steady-state mRNA levels determined by microarrays. This is not surprising, since chromatin structure only represents one mechanism of transcription regulation. In addition, transcription initiation is regulated by sequence-specific transcriptional activators and repressors. Steady-state mRNA levels are determined by the level of transcription, and post-transcriptional regulation of mRNA stability and degradation. Therefore, we would not expect chromatin structure to be the dominant determinant of steady-state mRNA levels.

The highly condensed heterochromatin that comprises most non-coding regions of higher eukaryotes represses transcription in those regions. However, in *P. falciparum*, a recent study found the repressive histone modification of H3K9me3 only at subtelomeric loci and not in other intergenic regions [43]. It may be that the generally relaxed, non-condensed structure of *P. falciparum *intergenic chromatin in core chromosomal regions is sufficiently permissive of transcription such that further relaxation is not required to recruit transcription factors and initiate high levels of transcription. Our Q-PCR validation at promoters of selected individual genes supported such a generalization (e.g., *var *genes, msp2). Another study found that the transcription pre-initiation complex was pre-assembled on promoters of all erythrocytic-expressed genes, independent of any histone acetylation [44], supporting this hypothesis, and challenging the model that chromatin acetylation precedes transcription initiation in *Plasmodium*.

In human cells, regulation of transcription by RNA Pol III requires histone acetylation and changes in chromatin condensation [45,46]. However, the consistently low nucleosomal occupancy of tRNA and 5S rRNA genes in *P. falciparum *may indicate a different mechanism for regulating Pol III-derived transcripts that may not involve interaction with modified histones but may instead depend on other Pol III associated transcription factors. Therefore, it may be advantageous to keep these genes in a perpetually permissive state to allow for rapid transcription of tRNAs when protein synthesis is rapidly accelerated in growing trophozoites. This same situation may apply to the RNA polymerase II transcribed genes that constitute the basal transcription machinery, which may need to be rapidly and highly expressed to initiate a general transcriptional increase in ring stage.

The expression of *var *genes is inversely correlated with nucleosomal occupancy. This has been confirmed from the dramatic changes in nucleosomal occupancy between active and silent var2CSA gene. The changes in nucleosomal occupancy observed at the intron of *var *genes supports the hypothesis that *var *gene regulation is based on cooperative interactions between the two promoters of each *var *gene [47]. The higher nucleosome occupancy in exon 2 of most *var *genes may inhibit full-length gene transcription compared with the lower nucleosomal occupancy in exon 1. This may provide an explanation for the low-level production of many *var *transcripts that are detectable by RT-PCR, but not detectable by Northern blots. The generally permissive chromatin structure of all *var *genes exon 1 may allow for abortive transcripts to be formed and rapidly degraded and for the generation of antisense transcript directed by the *var *intron. Transcription factor genes with patterns of nucleosome enrichment similar to VSAs may be involved in regulating the expression of these genes. The subnuclear localization of the singly expressed *var *gene may be shared with some of these similarly regulated core chromosomal genes.

The lack of histones in intergenic regions may prevent the condensation of chromosomes during late stages of mitotic replication, which does not occur in *Plasmodium *parasites as it does in other eukaryotic organisms. The telomeric regions display the highest chromatin condensation of any region of the genome. In other eukaryotes where chromosomes condense upon mitotic cell division, the centromere region is responsible for binding to the kinetochore and microtubules for chromosomal segregation in daughter cells. In *Plasmodium*, not all chromosomes have annotated centromeres, and the chromosomes do not condense at schizogony. The telomeric regions with TAREs and Rep20 that have higher nucleosomal enrichment may play a role in chromosome segregation into daughter cells [35]. This may also be an adaptation to the schizogony process where multiple rounds of nuclear replication precede cytokinesis in the development of merozoites. The highly repetitive genes that show telomere-like chromatin changes may be targeted by chromatin remodeling machinery that recognizes repetitive regions throughout the genome.

## Conclusions

*P. falciparum *exhibits unique chromatin structure due to its highly AT-rich genome, with histone-rich coding regions and relatively histone-free intergenic regions. Chromatin dynamics differ for coding, non-coding, subtelomeric and telomeric regions of the genome. Histone enrichment patterns allow for identification of new putative gene-coding regions. Numerous genes display dynamic changes in nucleosomal occupancy, but these changes do not correlate with mRNA levels for most genes. However, nucleosome enrichment at *var *genes is negatively correlated with expression. Our genome-wide nucleosome occupancy data provides a framework to build our understanding of the mechanisms of chromatin-mediated regulation of RNA expression and DNA replication throughout the cell cycle. Future studies will elaborate upon the role of specific histone modifications, and the interactions of nucleosomes with DNA binding proteins and transcription machinery to produce the highly regulated cycle of gene expression in this dangerous parasite.

## Methods

### Parasite culture

The *P. falciparum *3D7 clone was cultured at 5% hematocrit in type O^+ ^human red blood cells as previously described [48]. The parasites were synchronized twice by 5% sorbitol treatments at the ring stage [49]. To obtain parasite materials, infected erythrocytes were lysed by 0.1% saponin treatment, and parasite pellets were collected by centrifugation and washed twice with cold phosphate-buffered saline (PBS). Synchronized parasites were harvested at 12, 30 and 42 h to represent ring, trophozoite, and schizont stages, respectively.

### ChIP-chip

For ChIP, 3 × 10^9 ^trophozoite and schizont stage or 9 × 10^9 ^ring stage parasites were collected. First, the parasites were treated with 2 ml of permeabilization buffer (150 mM sucrose, 15 mM Tris-HCl, pH 7.5, 15 mM NaCl, 60 mM KCl, 2 mM CaCl_2_, 0.15 mM spermin, and 0.5 mM spermidine) for 5 min at room temperature. Micrococcal nuclease (MNase) digestion was performed at 37°C for 10 min using 10 units of the enzyme, which were titrated to digest most chromatin into mononucleosomes. The MNase digestion was stopped by 2 ml of stop solution (40 mM Tris-HCl, pH 8.0, 40 mM EDTA, 150 mM NaCl, 2% SDS, 1.2 mg/ml proteinase K) and incubating at 50°C for 15 min. After washing the parasites twice with cold PBS, the samples were crosslinked with 1% formaldehyde at room temperature for 30 min. After quenching the crosslinker with glycine, parasites were washed twice with cold PBS and resuspended in 1.5 ml of immunoprecipitation (IP) buffer (50 mM Hepes, pH 7.5, 140 mM NaCl, 1 mM EDTA, 1% Triton X-100, 0.1% sodium deoxycholate) with a protease inhibitor cocktail (Roche). A brief sonication was performed on ice for 20 s to break the cell membranes. After removing cell debris by centrifugation, the digested chromatin was used for IP with the anti-H4 antibodies (Millipore) as previously described [28]. Amplification of immunoprecipitated DNA was performed using a randomly primed PCR amplification method [50,51]. Fifty micrograms of amplified DNA were digested into 50 bp fragments in a 50 ml reaction with 2 units of DNase I (Promega) at 37°C for 1 min. After inactivating the enzyme at 95°C for 10 min, the digested DNA fragments were labeled with 1 mM Bio-N6-ddATP (Enzo) using 400 U terminal deoxynucleotidyl transferase (Roche) incubated for 1 hr at 37°C, then at 70°C for 10 minutes. ChIP with the anti-H3K9ac antibodies was performed similarly as with the anti-H4 antibodies.

### Hybridization and data analysis

End-labeled DNA was hybridized to the Affymetrix microarray in a hybridization mix prepared using the Affymetrix hybridization, Wash and Stain kit, following the manufacturer's instructions. Chips were washed on the Affymetrix Wash Station using a modified protocol Flex FS450_0001 with all temperatures set to 23°C. Chips were scanned on the Affymetrix scanner and data was analyzed and visualized using custom Matlab scripts available upon request. The array contains over 6.5 million probes total. 4,219,964 probes are uniquely mapped to a single location, and 4,642,958 probes mapped to 10 or fewer locations in the genome, with 91% of those uniquely mapped to a single location. Samples were normalized using median line normalization for invariant probes relative to two hybridizations of 3D7 genomic DNA (3D7-1, 3D7-2) described previously [52]. We calculated the log_2 _of the ratio of hybridization intensity of the test sample divided by the 3D7 reference for each probe. Then we calculated the running mean log_2 _ratio for windows of 150 bp for regions that contain at least 3 probes. For highly repetitive intergenic regions with no probe coverage, we connect the running mean line between the nearest two points with average log_2 _ratios. We compared the hybridization intensity of the ChIP-chip data with 3D7 genomic DNA data to determine genome-wide relative enrichment of nucleosomes. We compared the results of analyses using probes that uniquely mapped to a single location versus all probes that mapped to 10 or fewer locations in the genome. We found that inclusion of the probes mapped to 10 or fewer locations did not change the average nucleosome enrichment patterns for coding regions with good unique probe coverage (Table 1). This provided informative data for many subtelomeric multigene families with similar sequences. Therefore, we used this probe-mapping cutoff for all analyses. Our results demonstrated similar patterns of nucleosome enrichment for these multigene families, implying that the average signal distribution across the multiple locations is an accurate representation of the chromatin structure for these repetitive regions. The interpretation of an average log_2 _ratio over 150 bp also served to diminish the contribution of signal from outlier probes. Since chromatin structure is also influenced by DNA sequence, we expect similar nucleosomal occupancy signal from genes with similar sequences, and thus the probe signal will be similar at all locations. However, these results in identical repetitive telomeric and subtelomeric regions should be considered representative of the average nucleosomal occupancy of these sequences, and not precisely representing one particular region. Gene annotation information was taken from PlasmoDB version 5.5. Microarray data were submitted NCBI Gene Expression Omnibus (GEO) website with a provisional accession number GSE18968. Nucleosomal occupancy and the H3K9ac maps for *P. falciparum *chromosomes have also been submitted to PlasmoDB. Running median lines of log_2 _ratio for ring, trophozoite, and schizont are visible as Genome Browser tracks.

### Validation of the microarray data

To validate the microarray results, ChIP with anti-H4 antibodies followed by real-time PCR analysis for selected genes was performed using aliquots of the same parasite populations [28]. The relative nucleosome enrichment level of selected genes versus input DNA was calculated using 2^-ΔΔCt ^method with *MAL8P1.36 *as a reference gene which had stable nucleosome occupancy throughout IDC [53]. To determine whether the level of nucleosome occupancy is correlated with the expression levels of *var *genes, RNA was extracted from aliquots of the same parasite populations. Real-time RT-PCR was used to quantify the relative expression level of *var *genes using a set of primers to specifically amplify individual *var *genes with *seryl-tRNA synthetase *as a reference gene [54]. ChIP with anti-H4 antibodies followed by real-time PCR analysis was used to quantify the relative nucleosomal enrichment at *var *genes using the same set of primers with *MAL8P1.36 *as a reference. To further demonstrate that nucleosome occupancy is correlated with *var *gene expression, we panned the parasites with CSA to select parasites with *var2CSA *(*PFL0030c*) expression [55]. After three rounds of CSA selection, the expression level of each *var *gene was determined by real time RT-PCR to confirm that *var2CSA *was active. To compare the nucleosome occupancy of CSA-selected versus unselected parasites, ChIP and real-time PCR were performed using previously-described primers for different regions of *var2CSA *[56].

## List of Abbreviations

H4: histone H4; ChIP: chromatin immunoprecipitation; Q-PCR: quantitative polymerase chain reaction; RT-PCR: reverse-transcriptase polymerase chain reaction.

## Authors' contributions

SJW performed microarray hybridizations, analyzed the data and wrote the manuscript. Long Cui performed the ChIP, parasite panning and Q-PCR experiments and part of the writing. NVD provided bioinformatics support for analysis of microarray data. EAW provided advice on data analysis and helped to draft the manuscript. Liwang Cui conceived of the study, and helped to draft the manuscript. All authors read and approved of the final manuscript.

## Supplementary Material

Additional file 1 **A list of tables to show histone enrichment data at different regions of the genes**. Included are the mean log_2 _ratios of histone enrichment of all genes (S1), the exons (S2), putative transcription start sites (S3), the upstream intergenic minima (S4), and quantitative PCR validation of *var *gene expression and histone enrichment (S5). Click here for file

Additional file 2 **Box and whisker plot of ring stage probe log_2 _ratios by GC bin**. The distribution of probe log_2 _ratios for probes grouped into bins of increasing GC content for different regions of the genome. Columns represent 25mer probes with GC = 1-25. Y-axis is the average log2 ratio of ring stage H4 ChIP divided by genomic DNA hybridization intensity over a 150 bp window. The tops and bottoms of each "box" are the 25th and 75th percentiles of the samples. The red lines in the center of the box are the medians. The blue line represents a log2 ratio of zero. The high AT content of the *P. falciparum *genome results in very few probes with GC>20, thus producing empty columns for some of these bins. Click here for file

Additional file 3 **Numbers of probes in all GC bins **The numbers of probes mapped to 10 or fewer locations in A) the whole genome and B) regions with fewer probes, classified by the number of G or C nucleotides in the 25mer oligonucleotide probe. The highly AT-rich genome results in few probes with high GC content, and fewer probes in intergenic regions that are uniquely mapped. Click here for file

Additional file 4 **Non-subtelomeric genes with H3K9me3 modification enrichment**. Lines indicate average log_2 _ratio of H4 ChIP divided by genomic DNA hybridization intensity over 150 bp window, blue = ring, green = trophozoite, red = schizont. Blue (Red) genes are encoded on the top (bottom) strand. Click here for file

Additional file 5 **Core chromosomal genes with large changes in nucleosome enrichment**. Lines indicate average log_2 _ratio of H4 ChIP divided by genomic DNA hybridization intensity over 500 bp window, blue = ring, green = trophozoite, red = schizont. Blue (Red) genes are encoded on the top (bottom) strand. Click here for file

Additional file 6 **Genes with low nucleosomal occupancy at all times**. Lines indicate average log_2 _ratio of H4 ChIP divided by genomic DNA hybridization intensity over a 150 bp window: blue = ring, green = trophozoite, red = schizont. Blue (Red) genes are encoded on the top (bottom) strand. Click here for file

Additional file 7 **Genes with telomere-like nucleosome enrichment changes**. Lines indicate average log_2 _ratio of H4 ChIP divided by genomic DNA hybridization intensity over a 150 bp window: blue = ring, green = trophozoite, red = schizont. Blue (Red) genes are encoded on the top (bottom) strand. Click here for file

Additional file 8 **Genes with high nucleosome enrichment in rings, low in trophozoites and schizonts**. Lines indicate average log_2 _ratio of H4 ChIP divided by genomic DNA hybridization intensity over a 150 bp window: blue = ring, green = trophozoite, red = schizont. Blue (Red) genes are encoded on the top (bottom) strand. Click here for file

## References

[B1] Goldberg AD Allis CD Bernstein E Epigenetics: A landscape takes shape Cell cycle 2007 128 635 638 10.1016/j.cell.2007.02.006 17320500

[B2] Kouzarides T Chromatin modifications and their function Cell 2007 128 4 693 705 10.1016/j.cell.2007.02.005 17320507

[B3] Jenuwein T Allis CD Translating the histone code Science 293 5532 1074 1080 10.1126/science.1063127 11498575

[B4] Raisner RM Mahdhani HD Patterning chromatin: form and function for H2A.Z variant nucleosomes Cur Opin Gen Dev 2006 16 119 124 10.1016/j.gde.2006.02.005 16503125

[B5] Lieb JD Clarke ND Control of transcription through intragenic patterns of nucleosome composition Cell 2005 123 7 1187 1190 10.1016/j.cell.2005.12.010 16377560

[B6] Boeger H Griesenbeck J Strattan JS Kornberg RD Nucleosomes unfold completely at a transcriptionally active promoter Mol Cell 2003 11 6 1587 1598 10.1016/S1097-2765(03)00231-4 12820971

[B7] Henikoff S Nucleosomes at active promoters: unforgettable loss Cancer Cell 2007 12 407 409 10.1016/j.ccr.2007.10.024 17996642

[B8] Hogan GJ Lee CK Lieb JD Cell cycle-specified fluctuation of nucleosome occupancy at gene promoters PLoS Genet 2006 2 9 e158 10.1371/journal.pgen.0020158 17002501 PMC1570381

[B9] Schones DE Cui K Cuddapah S Roh TY Barski A Wang Z Wei G Zhao K Dynamic regulation of nucleosome positioning in the human genome Cell 2008 132 5 887 898 10.1016/j.cell.2008.02.022 18329373 PMC10894452

[B10] Bernstein BE Liu CL Humphrey EL Perlstein EO Schreiber SL Global nucleosome occupancy in yeast Genome Biol 2004 5 9 R62 10.1186/gb-2004-5-9-r62 15345046 PMC522869

[B11] Lee CK Shibata Y Rao B Strahl BD Lieb JD Evidence for nucleosome depletion at active regulatory regions genome-wide Nature Genet 2004 36 8 900 905 10.1038/ng1400 15247917

[B12] Yuan GC Liu YJ Dion MF Slack MD Wu LF Altschuler SJ Rando OJ Genome-scale identification of nucleosome positions in S. cerevisiae Science 2005 309 5734 626 630 10.1126/science.1112178 15961632

[B13] Lee W Tillo D Bray N Morse RH Davis RW Hughes TR Nislow C A high-resolution atlas of nucleosome occupancy in yeast Nature Genet 2007 39 10 1235 1244 10.1038/ng2117 17873876

[B14] Johnson SM Tan FJ McCullough HL Riordan DP Fire AZ Flexibility and constraint in the nucleosome core landscape of Caenorhabditis elegans chromatin Genome research 2006 16 12 1505 1516 10.1101/gr.5560806 17038564 PMC1665634

[B15] Valouev A Ichikawa J Tonthat T Stuart J Ranade S Peckham H Zeng K Malek JA Costa G McKernan K A high-resolution, nucleosome position map of C. elegans reveals a lack of universal sequence-dictated positioning Genome Res 2008 18 7 1051 1063 10.1101/gr.076463.108 18477713 PMC2493394

[B16] Mavrich TN Jiang C Ioshikhes IP Li X Venters BJ Zanton SJ Tomsho LP Qi J Glaser RL Schuster SC Nucleosome organization in the Drosophila genome Nature 2008 453 7193 358 362 10.1038/nature06929 18408708 PMC2735122

[B17] Liu X Lee CK Granek JA Clarke ND Lieb JD Whole-genome comparison of Leu3 binding in vitro and in vivo reveals the importance of nucleosome occupancy in target site selection Genome Res 2006 16 12 1517 1528 10.1101/gr.5655606 17053089 PMC1665635

[B18] Segal E Fondufe-Mittendorf Y Chen L Thastrom A Field Y Moore IK Wang JP Widom J A genomic code for nucleosome positioning Nature 2006 442 7104 772 778 10.1038/nature04979 16862119 PMC2623244

[B19] Ioshikhes IP Albert I Zanton SJ Pugh BF Nucleosome positions predicted through comparative genomics Nature Genet 2006 38 10 1210 1215 10.1038/ng1878 16964265

[B20] Gardner MJ Shallom SJ Carlton JM Salzberg SL Nene V Shoaibi A Ciecko A Lynn J Rizzo M Weaver B Sequence of *Plasmodium falciparum *chromosomes 2, 10, 11 and 14 Nature 2002 419 6906 531 534 10.1038/nature01094 12368868

[B21] Bozdech Z Llinas M Pulliam BL Wong ED Zhu J DeRisi JL The transcriptome of the intraerythrocytic developmental cycle of *Plasmodium falciparum *PLoS Biol 2003 1 1 E5 10.1371/journal.pbio.0000005 12929205 PMC176545

[B22] Le Roch KG Zhou Y Blair PL Grainger M Moch JK Haynes JD De La Vega P Holder AA Batalov S Carucci DJ Discovery of gene function by expression profiling of the malaria parasite life cycle Science 301 5639 1503 1508 10.1126/science.1087025 12893887

[B23] Aravind L Iyer LM Wellems TE Miller LH Plasmodium biology: genomic gleanings Cell 2003 115 7 771 785 10.1016/S0092-8674(03)01023-7 14697197

[B24] Cary C Lamont D Dalton JP Doerig C *Plasmodium falciparum *chromatin: nucleosomal organisation and histone-like proteins Parasitol Res 1994 80 3 255 258 10.1007/BF00932684 8036241

[B25] Miao J Fan Q Cui L Li J Li J Cui L The malaria parasite *Plasmodium falciparum *histones: organization, expression, and acetylation Gene 2006 369 53 65 10.1016/j.gene.2005.10.022 16410041

[B26] Chookajorn T Ponsuwanna P Cui L Mutually exclusive var gene expression in the malaria parasite: multiple layers of regulation Trends Parasitol 2008 24 10 455 461 10.1016/j.pt.2008.07.005 18771955

[B27] Cui L Fan Q Cui L Miao J Histone lysine methyltransferases and demethylases in *Plasmodium falciparum* Int J Parasitol 2008 38 10 1083 1097 10.1016/j.ijpara.2008.01.002 18299133 PMC4566933

[B28] Cui L Miao J Furuya T Li X Su XZ Cui L PfGCN5-mediated histone H3 acetylation plays a key role in gene expression in *Plasmodium falciparum* Eukaryot Cell 2007 6 7 1219 1227 10.1128/EC.00062-07 17449656 PMC1951105

[B29] Chookajorn T Dzikowski R Frank M Li F Jiwani AZ Hartl DL Deitsch KW Epigenetic memory at malaria virulence genes Proc Natl Acad Scie USA 2007 104 3 899 902 10.1073/pnas.0609084103 PMC1764221 17209011

[B30] Fan Q An L Cui L *Plasmodium falciparum *histone acetyltransferase, a yeast GCN5 homologue involved in chromatin remodeling Eukaryot Cell 2004 3 2 264 276 10.1128/EC.3.2.264-276.2004 15075257 PMC387650

[B31] Duraisingh MT Voss TS Marty AJ Duffy MF Good RT Thompson JK Freitas-Junior LH Scherf A Crabb BS Cowman AF Heterochromatin silencing and locus repositioning linked to regulation of virulence genes in *Plasmodium falciparum* Cell 2005 121 1 13 24 10.1016/j.cell.2005.01.036 15820675

[B32] Freitas-Junior LH Hernandez-Rivas R Ralph SA Montiel-Condado D Ruvalcaba-Salazar OK Rojas-Meza AP Mancio-Silva L Leal-Silvestre RJ Gontijo AM Shorte S Telomeric heterochromatin propagation and histone acetylation control mutually exclusive expression of antigenic variation genes in malaria parasites Cell 2005 121 1 25 36 10.1016/j.cell.2005.01.037 15820676

[B33] Corcoran LM Thompson JK Walliker D Kemp DJ Homologous recombination within subtelomeric repeat sequences generates chromosome size polymorphisms in *P. falciparum* Cell 1988 53 5 807 813 10.1016/0092-8674(88)90097-9 3286016

[B34] Figueiredo LM Pirrit LA Scherf A Genomic organisation and chromatin structure of *Plasmodium falciparum *chromosome ends Mol Biochem Parasitol 2000 106 1 169 174 10.1016/S0166-6851(99)00199-1 10743621

[B35] O'Donnell RA Freitas-Junior LH Preiser PR Williamson DH Duraisingh M McElwain TF Scherf A Cowman AF Crabb BS A genetic screen for improved plasmid segregation reveals a role for Rep20 in the interaction of *Plasmodium falciparum *chromosomes EMBO J 2002 21 5 1231 1239 10.1093/emboj/21.5.1231 11867551 PMC125903

[B36] Watanabe J Wakaguri H Sasaki M Suzuki Y Sugano S Comparasite: a database for comparative study of transcriptomes of parasites defined by full-length cDNAs Nucleic Acids Res 2007 35 D431 438 10.1093/nar/gkl1039 17151081 PMC1781114

[B37] Zhou Y Ramachandran V Kumar KA Westenberger S Refour P Zhou B Li F Young JA Chen K Plouffe D Evidence-Based Annotation of the Malaria Parasite's Genome Using Comparative Expression Profiling PLoS ONE 2008 3 2 e1570 10.1371/journal.pone.0001570 18270564 PMC2215772

[B38] Salcedo-Amaya AM van Driel MA Alako BT Trelle MB Elzen AM van den Cohen AM Janssen-Megens EM Vegte-Bolmer M van de Selzer RR Iniguez AL Dynamic histone H3 epigenome marking during the intraerythrocytic cycle of *Plasmodium falciparum* Proc Natl Acad Scie USA 2009 106 24 9655 9660 10.1073/pnas.0902515106 PMC2701018 19497874

[B39] Haran TE Mohanty U The unique structure of A-tracts and intrinsic DNA bending Q Rev Biophys 2009 42 1 41 81 10.1017/S0033583509004752 19508739

[B40] Suter B Schnappauf G Thoma F Poly(dA.dT) sequences exist as rigid DNA structures in nucleosome-free yeast promoters in vivo Nucleic Acids Res 2000 28 21 4083 4089 10.1093/nar/28.21.4083 11058103 PMC113125

[B41] Yaragatti M Basilico C Dailey L Identification of active transcriptional regulatory modules by the functional assay of DNA from nucleosome-free regions Genome Res 2008 18 6 930 938 10.1101/gr.073460.107 18441229 PMC2413160

[B42] Watanabe J Sasaki M Suzuki Y Sugano S Analysis of transcriptomes of human malaria parasite *Plasmodium falciparum *using full-length enriched library: identification of novel genes and diverse transcription start sites of messenger RNAs Gene 2002 291 1-2 105 113 10.1016/S0378-1119(02)00552-8 12095684

[B43] Lopez-Rubio JJ Mancio-Silva L Scherf A Genome-wide analysis of heterochromatin associates clonally variant gene regulation with perinuclear repressive centers in malaria parasites Cell Host Microbe 2009 5 2 179 190 10.1016/j.chom.2008.12.012 19218088

[B44] Gopalakrishnan AM Nyindodo LA Ross Fergus M Lopez-Estrano C *Plasmodium falciparum*: Preinitiation complex occupancy of active and inactive promoters during erythrocytic stage Exp Parasitol 2009 121 1 46 54 10.1016/j.exppara.2008.09.016 18951895

[B45] Kenneth NS Ramsbottom BA Gomez-Roman N Marshall L Cole PA White RJ TRRAP and GCN5 are used by c-Myc to activate RNA polymerase III transcription Proc Natl Acad Scie USA 2007 104 38 14917 14922 10.1073/pnas.0702909104 PMC1986588 17848523

[B46] Tse C Sera T Wolffe AP Hansen JC Disruption of higher-order folding by core histone acetylation dramatically enhances transcription of nucleosomal arrays by RNA polymerase III Mol Cell Biol 1998 18 8 4629 4638 9671473 10.1128/mcb.18.8.4629 PMC109049

[B47] Dzikowski R Li F Amulic B Eisberg A Frank M Patel S Wellems TE Deitsch KW Mechanisms underlying mutually exclusive expression of virulence genes by malaria parasites EMBO Rep 2007 8 10 959 965 10.1038/sj.embor.7401063 17762879 PMC2002552

[B48] Trager W Jensen JB Human malaria parasites in continuous culture Science 1976 193 4254 673 675 10.1126/science.781840 781840

[B49] Lambros C Vanderberg JP Synchronization of *Plasmodium falciparum *erythrocytic stages in culture J Parasitol 1979 65 3 418 420 10.2307/3280287 383936

[B50] Lieb JD Liu X Botstein D Brown PO Promoter-specific binding of Rap1 revealed by genome-wide maps of protein-DNA association Nat Genet 2001 28 4 327 334 10.1038/ng569 11455386

[B51] Schubeler D Scalzo D Kooperberg C van Steensel B Delrow J Groudine M Genome-wide DNA replication profile for Drosophila melanogaster: a link between transcription and replication timing Nat Genet 2002 32 3 438 442 10.1038/ng1005 12355067

[B52] Dharia NV Sidhu AB Cassera MB Westenberger SJ Bopp SE Eastman RT Plouffe D Batalov S Park DJ Volkman SK Use of high-density tiling microarrays to globally identify mutations and elucidate mechanisms of drug resistance in *Plasmodium falciparum* Genome Biol 2009 10 2 R21 10.1186/gb-2009-10-2-r21 19216790 PMC2688282

[B53] Livak KJ Schmittgen TD Analysis of relative gene expression data using real-time quantitative PCR and the 2^-ΔΔCt ^method Methods 2001 25 4 402 408 10.1006/meth.2001.1262 11846609

[B54] Salanti A Staalsoe T Lavstsen T Jensen AT Sowa MP Arnot DE Hviid L Theander TG Selective upregulation of a single distinctly structured var gene in chondroitin sulphate A-adhering *Plasmodium falciparum *involved in pregnancy-associated malaria Mol Microbiol 2003 49 1 179 191 10.1046/j.1365-2958.2003.03570.x 12823820

[B55] Alkhalil A Achur RN Valiyaveettil M Ockenhouse CF Gowda DC Structural requirements for the adherence of *Plasmodium falciparum *-infected erythrocytes to chondroitin sulfate proteoglycans of human placenta J Biol Chem 2000 275 51 40357 40364 10.1074/jbc.M006399200 11005815

[B56] Lopez-Rubio JJ Riviere L Scherf A Shared epigenetic mechanisms control virulence factors in protozoan parasites Cur Opin Microbiol 2007 10 6 560 568 10.1016/j.mib.2007.10.003 18024150

