# Supplementary figures and images for: Genome-wide nucleosome mapping of Plasmodium falciparum reveals histone-rich coding and histone-poor intergenic regions and chromatin remodeling of core and subtelomeric genes
Source: BMC Genomics. 2009 Dec 16;10:610. doi: 10.1186/1471-2164-10-610 (PMC2801526; doi:10.1186/1471-2164-10-610)

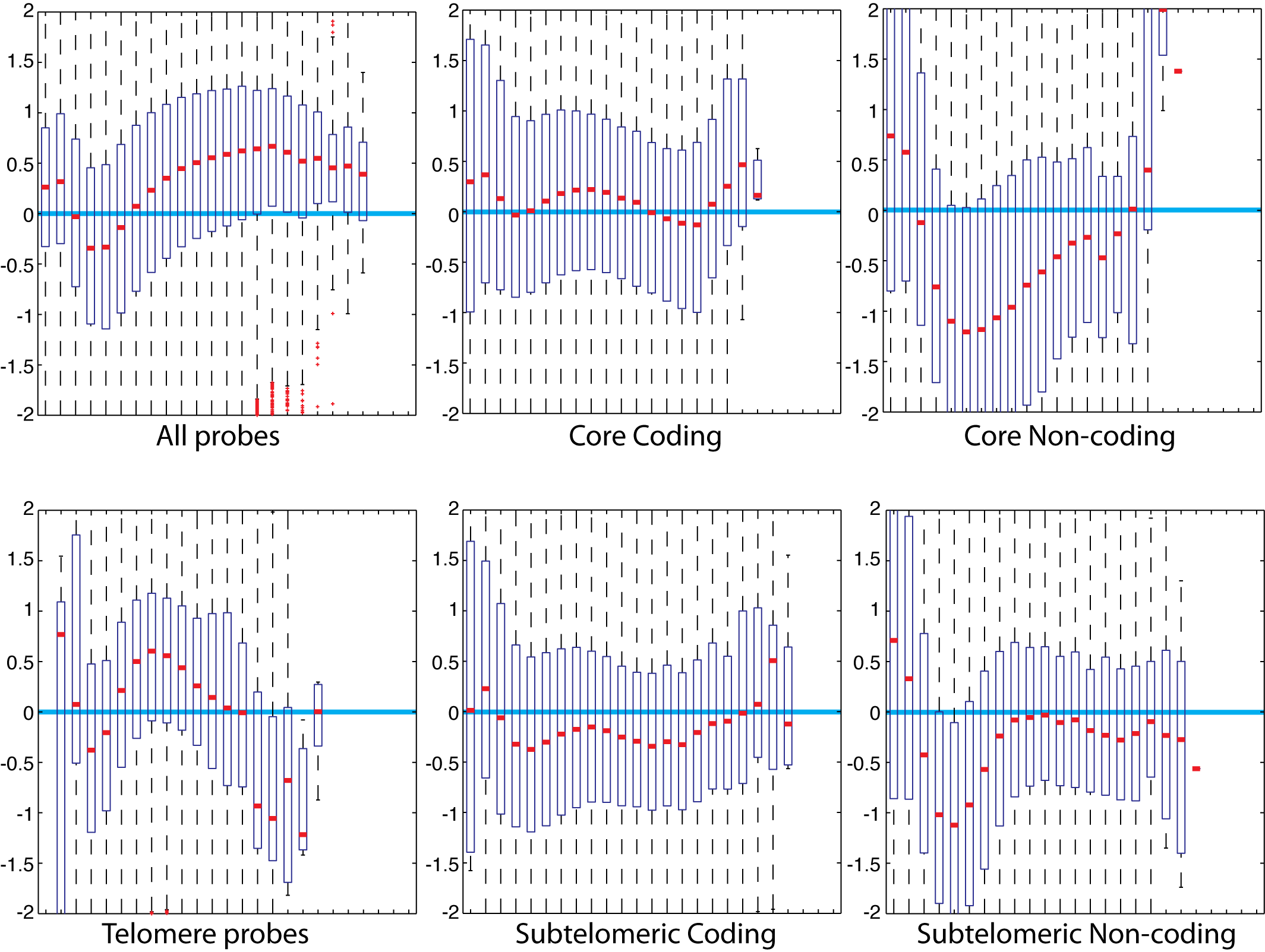

Supplement: Additional file 2 — Box and whisker plot of ring stage probe log2 ratios by GC bin. The distribution of probe log2 ratios for probes grouped into bins of increasing GC content for different regions of the genome. Columns represent 25mer probes with GC = 1-25. Y-axis is the average log2 ratio of ring stage H4 ChIP divided by genomic DNA hybridization intensity over a 150 bp window. The tops and bottoms of each "box" are the 25th and 75th percentiles of the samples. The red lines in the center of the box are the medians. The blue line represents a log2 ratio of zero. The high AT content of the P. falciparum genome results in very few probes with GC>20, thus producing empty columns for some of these bins. [file 1471-2164-10-610-S2.PNG]

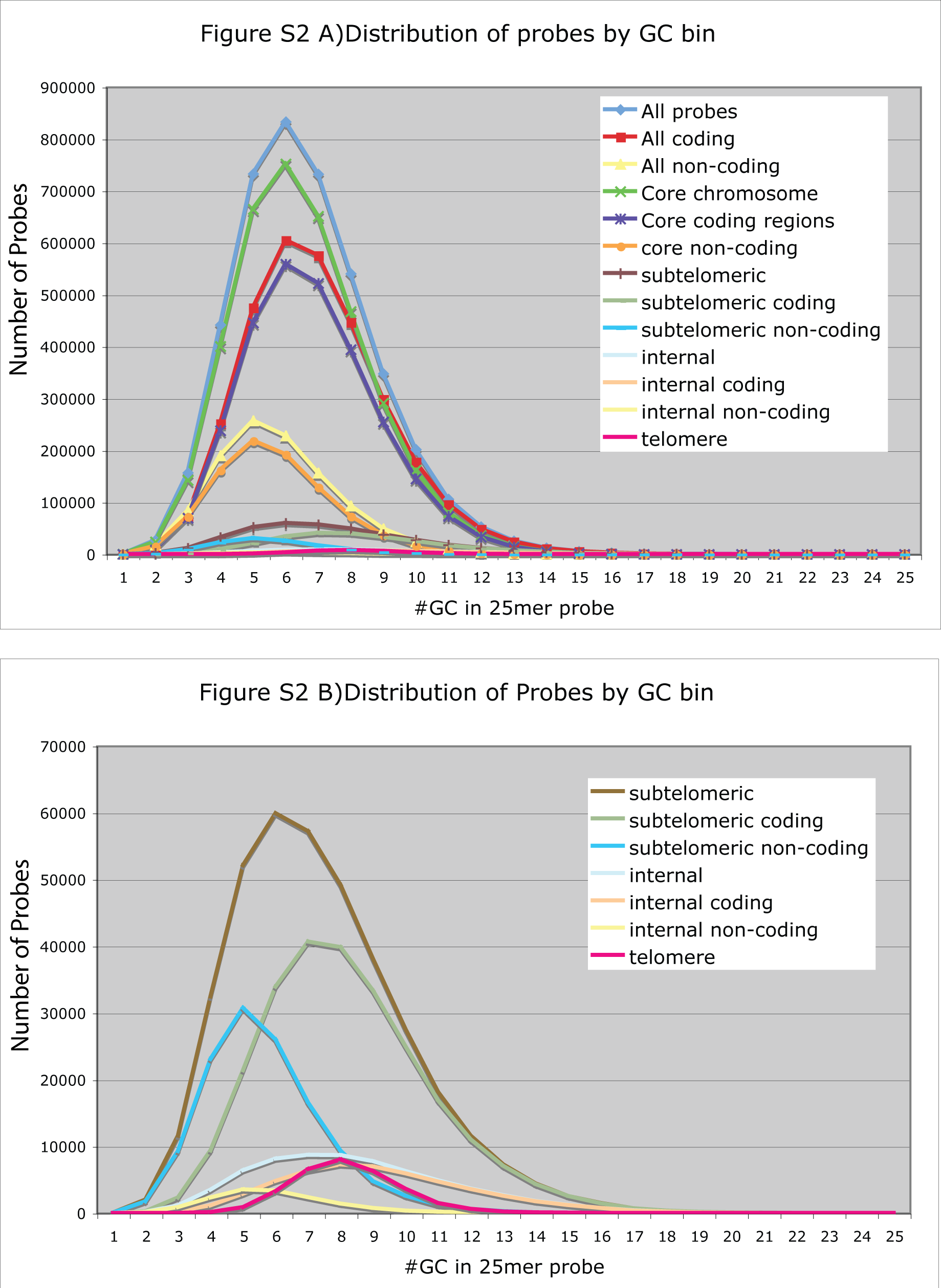

Supplement: Additional file 3 — Numbers of probes in all GC bins The numbers of probes mapped to 10 or fewer locations in A) the whole genome and B) regions with fewer probes, classified by the number of G or C nucleotides in the 25mer oligonucleotide probe. The highly AT-rich genome results in few probes with high GC content, and fewer probes in intergenic regions that are uniquely mapped. [file 1471-2164-10-610-S3.PNG]

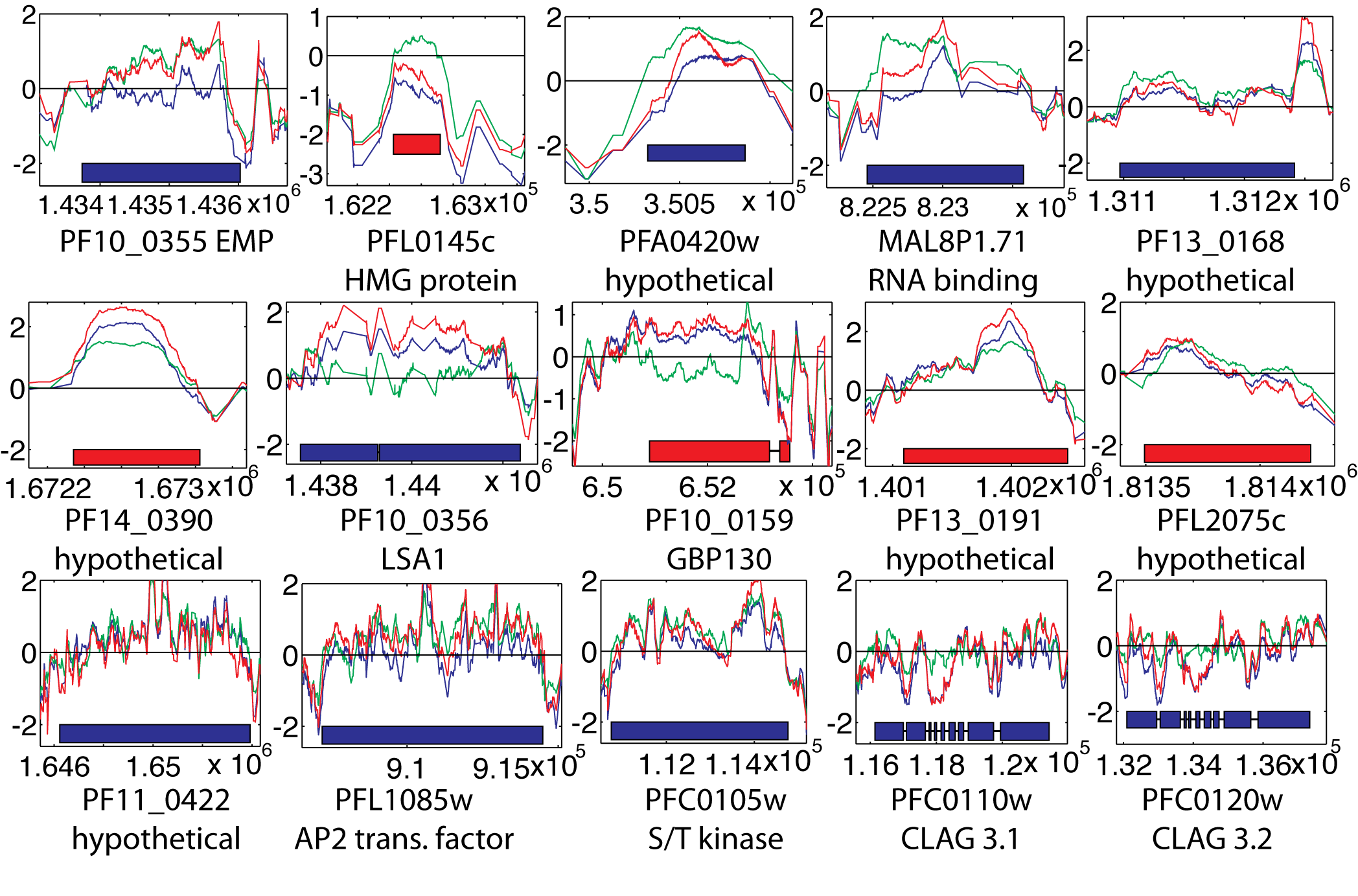

Supplement: Additional file 4 — Non-subtelomeric genes with H3K9me3 modification enrichment. Lines indicate average log2 ratio of H4 ChIP divided by genomic DNA hybridization intensity over 150 bp window, blue = ring, green = trophozoite, red = schizont. Blue (Red) genes are encoded on the top (bottom) strand. [file 1471-2164-10-610-S4.PNG]

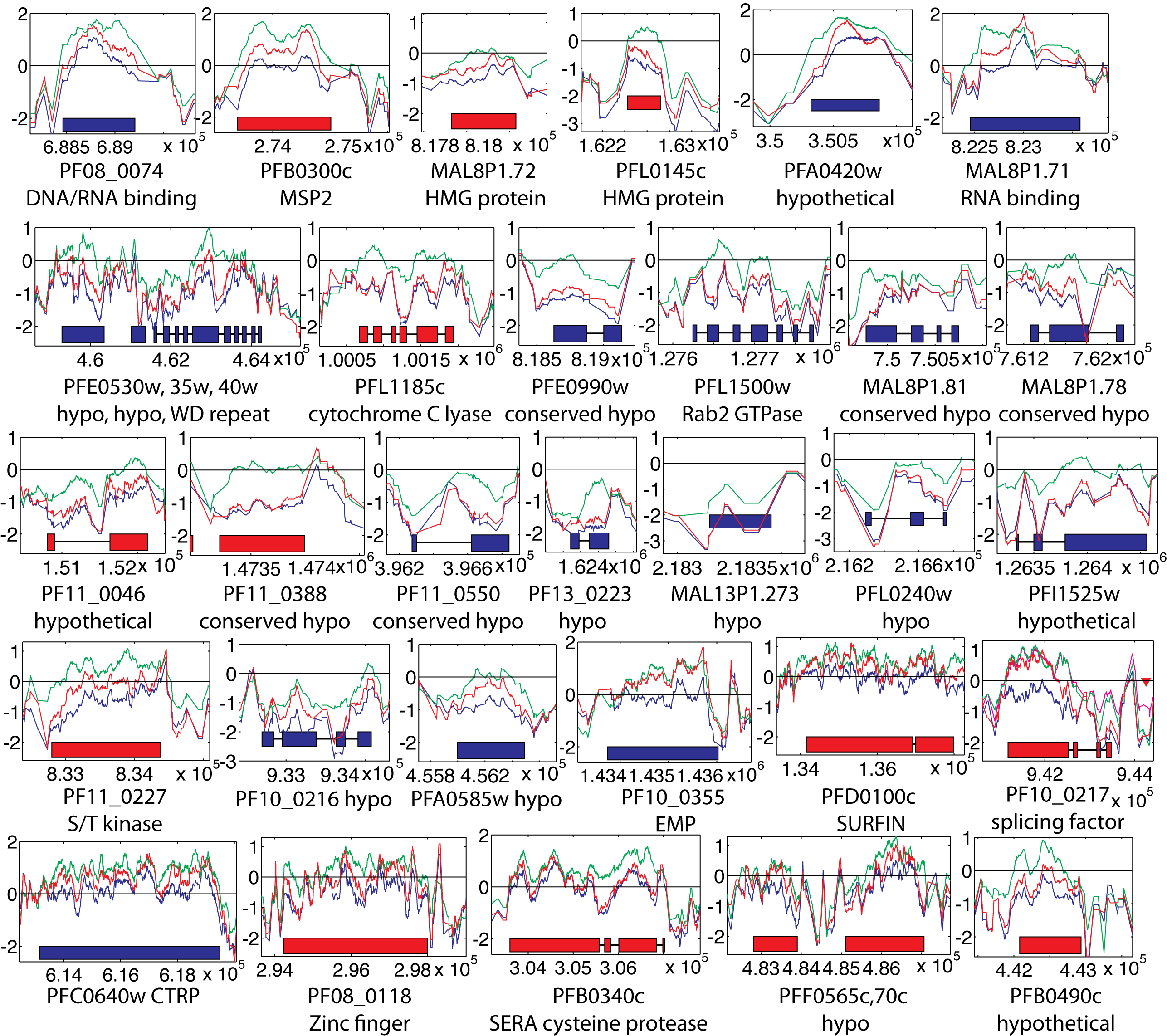

Supplement: Additional file 5 — Core chromosomal genes with large changes in nucleosome enrichment. Lines indicate average log2 ratio of H4 ChIP divided by genomic DNA hybridization intensity over 500 bp window, blue = ring, green = trophozoite, red = schizont. Blue (Red) genes are encoded on the top (bottom) strand. [file 1471-2164-10-610-S5.PNG]

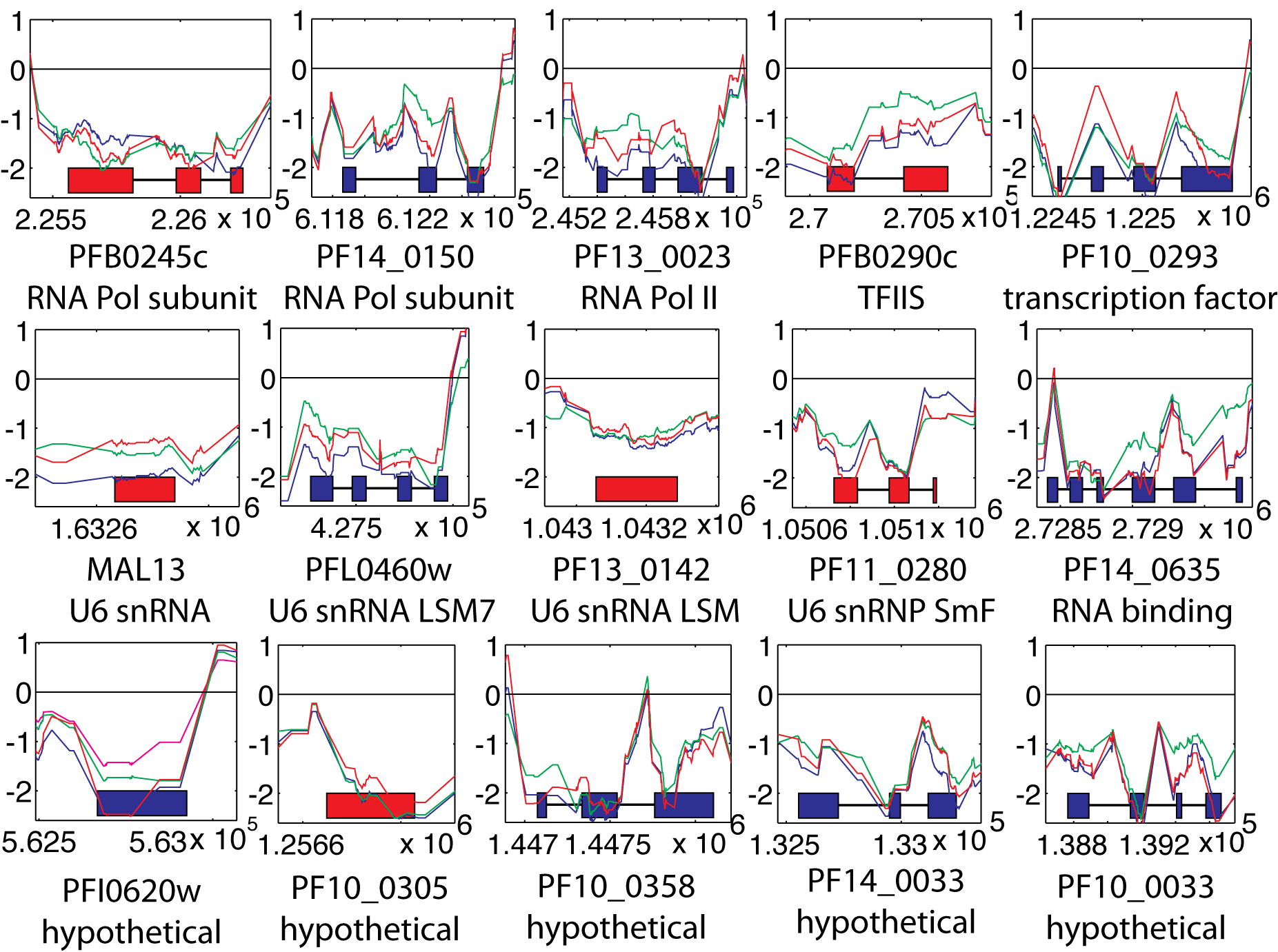

Supplement: Additional file 6 — Genes with low nucleosomal occupancy at all times. Lines indicate average log2 ratio of H4 ChIP divided by genomic DNA hybridization intensity over a 150 bp window: blue = ring, green = trophozoite, red = schizont. Blue (Red) genes are encoded on the top (bottom) strand. [file 1471-2164-10-610-S6.PNG]

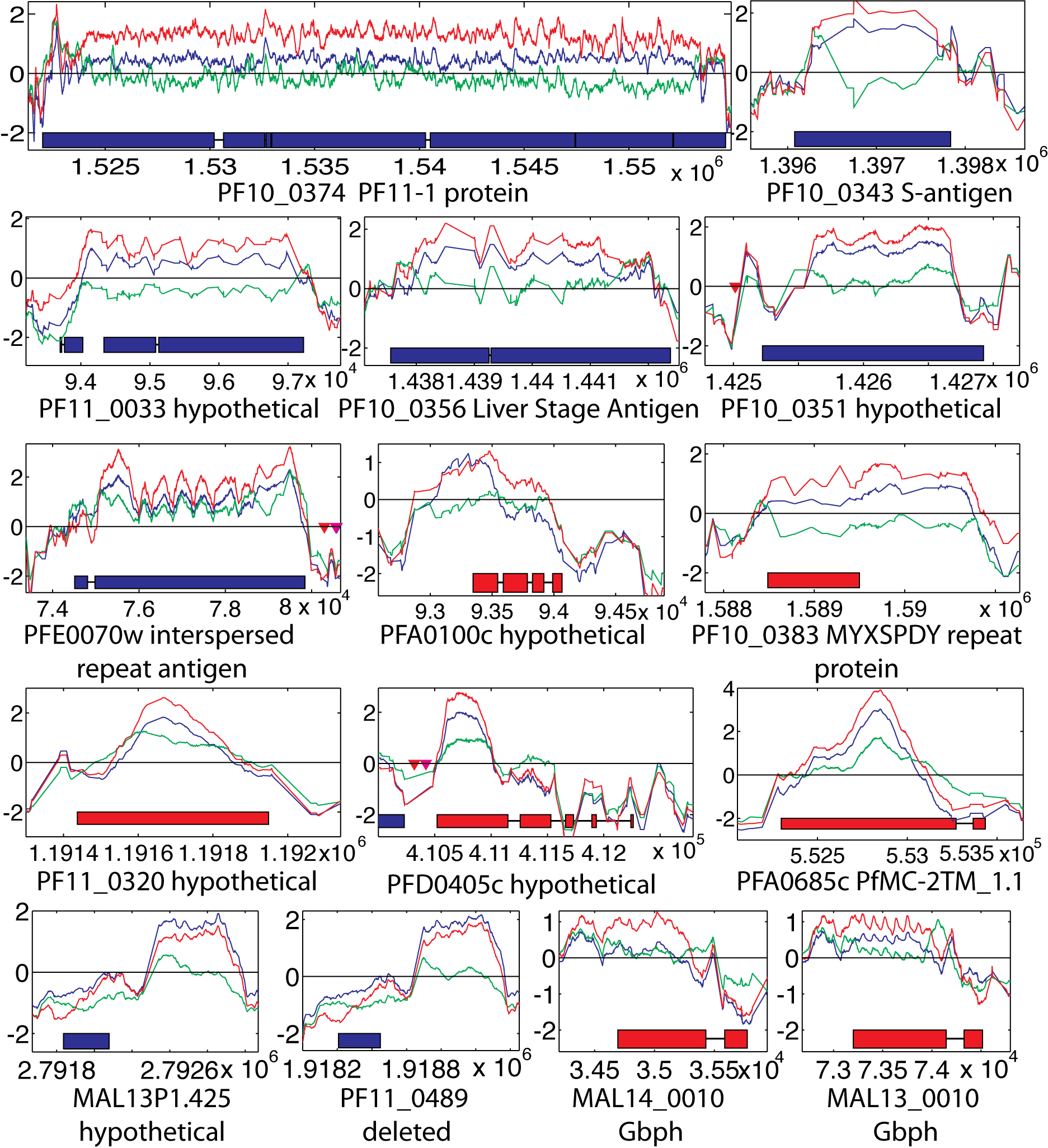

Supplement: Additional file 7 — Genes with telomere-like nucleosome enrichment changes. Lines indicate average log2 ratio of H4 ChIP divided by genomic DNA hybridization intensity over a 150 bp window: blue = ring, green = trophozoite, red = schizont. Blue (Red) genes are encoded on the top (bottom) strand. [file 1471-2164-10-610-S7.PNG]

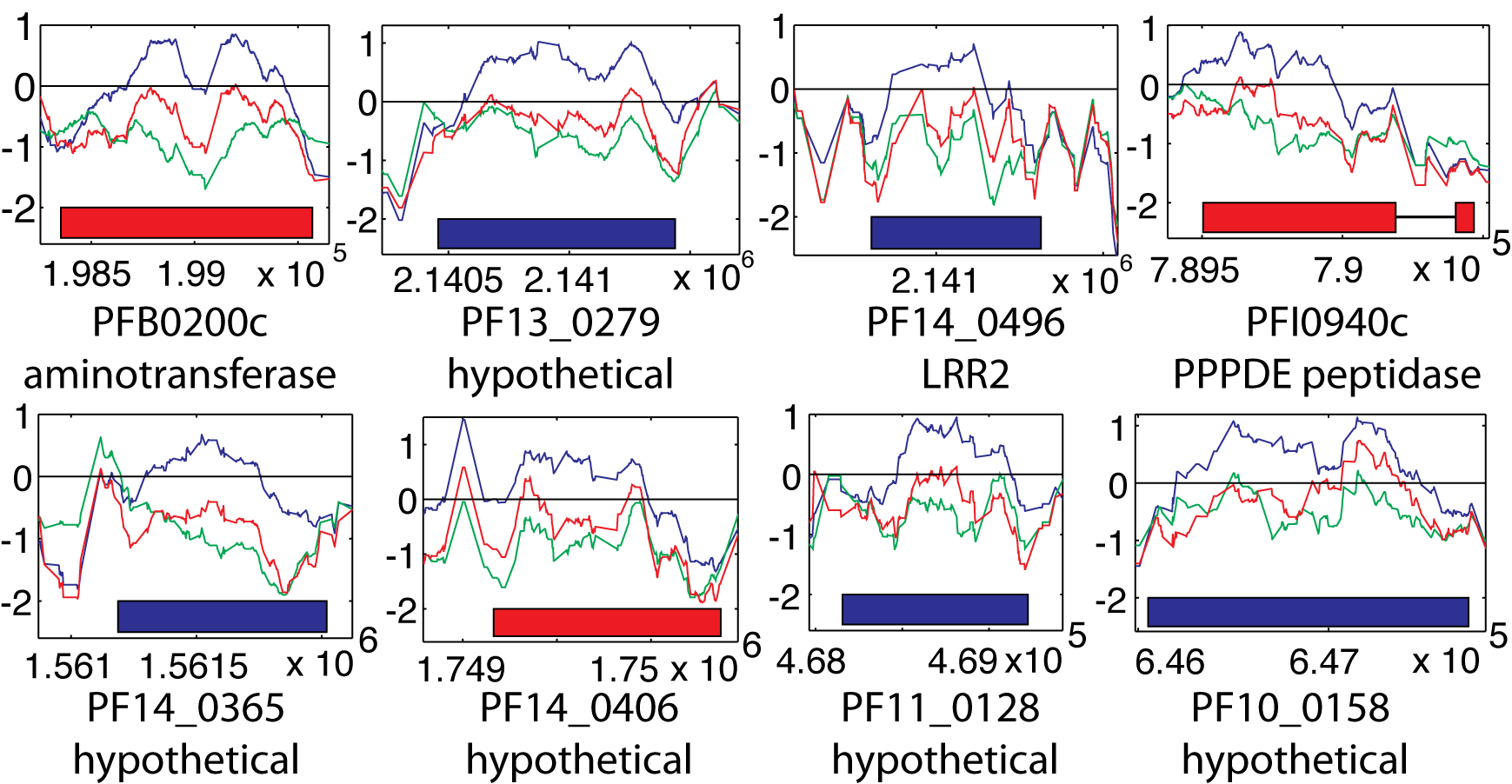

Supplement: Additional file 8 — Genes with high nucleosome enrichment in rings, low in trophozoites and schizonts. Lines indicate average log2 ratio of H4 ChIP divided by genomic DNA hybridization intensity over a 150 bp window: blue = ring, green = trophozoite, red = schizont. Blue (Red) genes are encoded on the top (bottom) strand. [file 1471-2164-10-610-S8.PNG]
